# *Helicobacter pylori* Usurps Cell Polarity to Turn the Cell Surface into a Replicative Niche

**DOI:** 10.1371/journal.ppat.1000407

**Published:** 2009-05-01

**Authors:** Shumin Tan, Lucy S. Tompkins, Manuel R. Amieva

**Affiliations:** 1 Department of Microbiology and Immunology, Stanford University, Stanford, California, United States of America; 2 Department of Medicine, Division of Infectious Diseases and Geographic Medicine, Stanford University, Stanford, California, United States of America; 3 Department of Pediatrics, Stanford University, Stanford, California, United States of America; Institut Pasteur, France

## Abstract

*Helicobacter pylori* (*Hp*) intimately interacts with the gastric epithelial surface and translocates the virulence factor CagA into host cells in a contact-dependent manner. To study how *Hp* benefits from interacting with the cell surface, we developed live-cell microscopy methods to follow the fate of individual bacteria on the cell surface and find that *Hp* is able to replicate and form microcolonies directly over the intercellular junctions. On polarized epithelia, *Hp* is able to grow directly on the apical cell surface in conditions that do not support the growth of free-swimming bacteria. In contrast, mutants in CagA delivery are defective in colonization of the apical cell surface. *Hp* perturbs the polarized epithelium in a highly localized manner, since wild-type *Hp* does not rescue the growth defect of the CagA-deficient mutants upon co-infection. CagA's ability to disrupt host cell polarity is a key factor in enabling colonization of the apical cell surface by *Hp*, as disruption of the atypical protein kinase C/Par1b polarity pathway leads to rescue of the mutant growth defect during apical infection, and CagA-deficient mutants are able to colonize the polarized epithelium when given access to the basolateral cell surface. Our study establishes the cell surface as a replicative niche and the importance of CagA and its effects on host cell polarity for this purpose.

## Author Summary

*Helicobacter pylori* (*Hp*) is a bacterium that chronically infects the human stomach, in some cases leading to diseases such as stomach cancer and ulcers. The bacteria live in close proximity to the epithelial lining and can adhere directly to the host cell membrane and deliver toxins. We utilized live-cell imaging and a cell culture model of polarized epithelial cells to address why *Hp* attaches to the cell surface. We discovered that *Hp* is able to grow on the surface of epithelial cells, even in conditions where the free-swimming bacteria are rapidly killed. One mechanism involved in this ability to colonize the cell surface is the virulence factor CagA, which is injected directly into host cells by the bacteria. We found that CagA's ability to perturb cell polarity is important for the efficient survival and growth of *Hp* on the apical surface of the host cell. Our study establishes the cell surface as a niche for bacterial growth and elucidates a role of CagA for the bacterium.

## Introduction

In order to cause infection bacteria have to colonize a specialized niche within the host. This implies adaptations that allow bacteria to reach, extract nutrients from, and replicate within particular anatomical and physiological environments in the host. Bacteria that colonize mucosal surfaces, for example, have to reach the apical surface of polarized epithelial cells in the face of mechanical and chemical clearance mechanisms, attach to molecules tethered to the cell membrane, and extract nutrients from the immediate environment to persist and replicate. Some mucosal colonizers replicate near the cell surface within epithelial secretions [1], while others are found directly adhered to the apical surface of the cells, forming clusters of bacteria that suggest replication on the cell surface [2]–[6]. How cell surface-adhered bacteria affect the host cell in order to survive and replicate in this niche has not been well studied.

*Helicobacter pylori* (*Hp*) represents an important example of a bacterium that establishes long-term and intimate interactions with the epithelial surface, in some cases causing serious diseases such as peptic ulcers and gastric cancer [7]. Rather than persisting in the harsh environment of the stomach lumen, *Hp* has evolved the capacity to avoid the microbicidal acid in the stomach, by reaching and colonizing a very narrow anatomical niche near the surface of the epithelial lining. All *Hp* are found within 25 µm of the cell surface in the mucus layer immediately overlaying the cells [8]. Within this microenvironment, *Hp* survives as two major populations: one that is free-swimming in the mucus gel and utilizes motility, chemotaxis and stress responses to survive and swim towards the shelter of the epithelium; and a second population (∼20%) found directly adhered to the epithelial surface via multiple adhesins [9]–[11]. The more virulent strains of *Hp* also have contact-dependent mechanisms to interact with and modify epithelial cells, including a type IV secretion system (TFSS) that injects the virulence factor CagA into host cells [12]–[16]. CagA has multiple effects on epithelial cells, including the potential to modify apical junctions and perturb cell polarity, but its function for the benefit of the bacteria has not been established [17]–[21].

We hypothesized that *Hp* has evolved the capacity to adhere to and deliver bacterial products to the epithelial cells in order to colonize and grow directly on the cell surface, independently of the free-swimming form. We thus asked (i) whether *Hp* can replicate directly on the epithelial cell membrane, (ii) whether *Hp* can usurp the barrier and polarity of the epithelium to obtain nutrients from the cells or the interstitium in conditions where nutrients for free-swimming bacteria are limited, and (iii) whether the cag TFSS and CagA play a role in colonization of the cell surface.

In this study we show, in a cell culture model of a polarized epithelium, that *Hp* is able to replicate and grow directly on the cell surface in conditions that do not support the growth of the free-swimming bacteria. We also show that the contact-dependent virulence factor CagA plays a key role in altering epithelial cell polarity to enable adhered *Hp* to turn the apical cell surface into a replicative niche.

## Results

### *Hp* replicate and form clonal microcolonies directly at epithelial intercellular junctions

**Figure 1:**
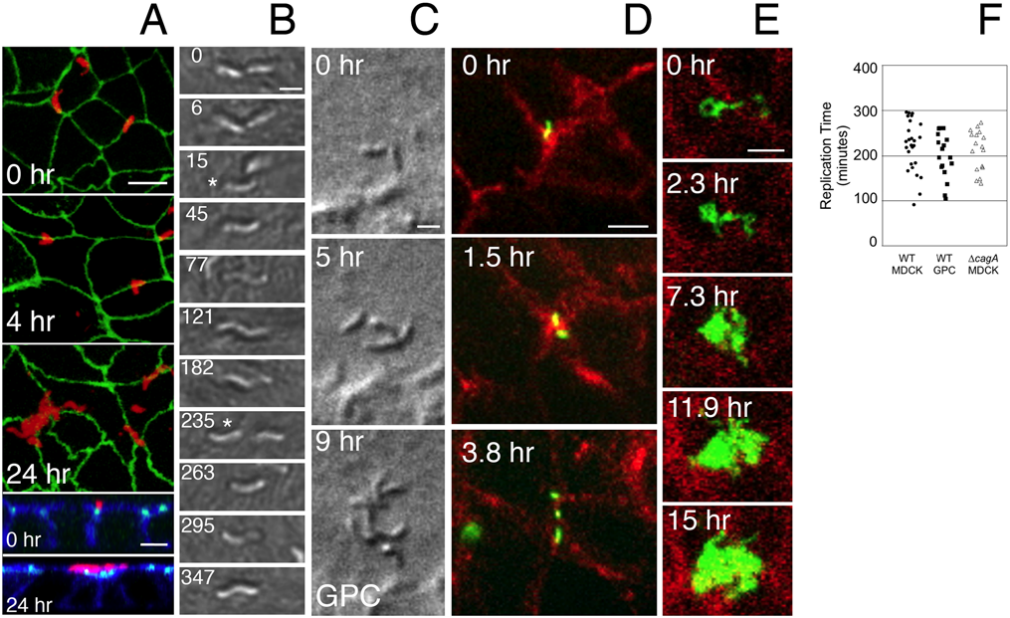
*Hp* replicate on the cell surface. (A) Confocal immunofluorescence time course of *Hp* attachment and microcolony formation on the cell surface. MDCK cells were infected for 5 minutes and washed (0 hr), and then incubated for times shown. Monolayers were stained with anti-*Hp* antibodies (red) and for the apical junctions (anti-ZO-1, green). Bottom panels show cross sections through the monolayer (also stained for f-actin in blue). Scale bars 5 µm. (B) DIC time-lapse of *Hp* replicating on the MDCK cell surface. Single bacteria were followed through 1.5 rounds of replication. Star indicates the bacterium of each pair being followed. Images from Video S1 aligned horizontally. Time in minutes is indicated. Scale bar 1 µm. (C) DIC time-lapse movie of *Hp* replicating on the cell surface of primary murine gastric epithelial cells (GPC) of pit lineage. *Hp* were attached near a cell junction at 0 hr and followed through 2 rounds of replication (see Video S2). Scale bar 1 µm. (D) Time-lapse fluorescence microscopy of GFP-*Hp* (green) replicating over the epithelial junctions of MDCK cells expressing RFP-E-cadherin (red). GFP-*Hp* shown in the top panel was filmed as it adhered to the cell surface, and followed for several hours (see Video S3). Scale bar 5 µm. (E) A longer time-lapse recording of cells infected as in (D), starting a day after initial infection (see Video S4). Scale bar 5 µm. (F) *Hp* replication rate on the surface of MDCK or Gastric Primary Cells (GPC). *Hp* were followed by DIC microscopy as in (B) and (C). Cycles of replication were noted by single frame analysis of the video recordings taken at 1–2 minute resolution. Points on the graph represent individual *Hp* that were followed over a replication cycle, collected from multiple experiments. Median rates of replication on the cell surface were 227, 196, and 220 minutes for WT on MDCK, WT on GPC, and Ä*cagA* on MDCK respectively. These were not significantly different from each other as determined by Mann-Whitney statistical test.

During initial interaction with an epithelium, *Hp* preferentially adhere to the intercellular junctions as single spiral bacteria (Figure 1A) [18]. Over a period of 12 to 24 hours, clusters comprised of 8 or more *Hp* become visible over the intercellular junctions (Figure 1A), suggesting that initially attached *Hp* replicate to form microcolonies. However, static images from a time-course cannot distinguish between clonal growth of microcolonies vs. aggregation of adhered bacteria into clusters, or the recruitment of new free-swimming bacteria to sites of adhesion. To directly distinguish between these possibilities, we developed a live-cell imaging system controlled for temperature, atmosphere and light levels, to enable tracking of individual *Hp* after attachment to the cell surface (see Materials and Methods for details). We observed individual *Hp* elongating and dividing, over several cycles of replication, all while remaining adhered to the cell membrane near their original attachment site (Figure 1B; Video S1).

**Figure 2:**
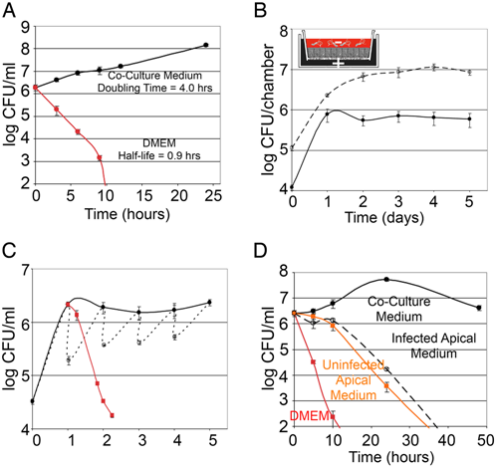
*Hp* grows on a polarized epithelium in conditions where free-swimming bacteria are killed. (A) *Hp* growth in broth. *Hp* grown on blood agar plates were resuspended and inoculated into DMEM with FBS and Brucella broth (co-culture media) (black line) or into DMEM only (red line). Samples were taken over time and plated for colony forming unit (CFU) counts. (B–C) *Hp* growth in a Transwell system. To mimic tissue polarity during infection, polarized epithelial cells were grown on Transwell chambers as shown in the diagram. Co-culture media able to support the growth of cells and bacteria was placed only in the basal chamber (+). *Hp* were inoculated into the apical chamber in DMEM (−). (B) Bacterial counts of the cell-associated *Hp* population (dashed line), and simultaneously of the free-swimming population (solid line). (C) Bacteria counts from the free-swimming population sampled before media replacement daily (solid line), and counts before and after media change daily (dotted line). The red plot indicates counts from the free-swimming population removed from the apical chamber at day 1 post-infection, and incubated without host cells. (D) Conditioned media from the apical chamber does not support *Hp* growth. The media from the apical chamber was collected after 1 day of infection or from uninfected polarized monolayers, filtered, and then inoculated with plate-grown bacteria. As positive and negative controls, co-culture media or DMEM were inoculated with the same bacterial suspension. Samples were taken over time and plated for CFU counts.

The median doubling time of *Hp* on MDCK cells was 4 hours, similar to that of a *Hp* population grown in standard broth (planktonic) culture (Figure 1F and Figure 2A). We found similar replication on the surface of AGS cells (derived from a human gastric adenocarcinoma), and on Caco-2 cells (human colon carcinoma cells) (not shown). We also found that *Hp* can infect primary murine gastric epithelial cells, forming clonal microcolonies on the cell surface with a replication rate similar to that on MDCK cells (Figure 1C and 1F, Video S2) [22],[23].

To better visualize the spatial relationship between the epithelial junctions and the replicating cell-associated *Hp*, we infected MDCK cells stably expressing the junctional protein E-cadherin fused to red fluorescent protein (MDCK Ecad-RFP), with *Hp* expressing green fluorescent protein (GFP). We confirmed that individual *Hp* preferentially adhere over the junctional area, and remain there as they replicate, even as the host cells move (Figure 1D, Video S3). This persisted throughout the course of 2 days, resulting in the formation of large microcolonies (Figure 1E, Video S4).

### *Hp* grow on the apical surface of a polarized monolayer in conditions where free-swimming bacteria are killed

The finding that *Hp* adhere to and replicate directly on the cell surface suggests that *Hp* has evolved specific adaptations to survive on this niche. However, *in vivo*, cell polarity allows epithelial cells to create distinct apical (luminal) vs. basolateral (interstitial) cell surfaces. The apical surface of epithelia is bathed in conditions very different from those of the interstitial compartment, which contains nutrients necessary for epithelial maintenance [24]. To better mimic intact tissue polarity, we used a Transwell filter system that allows tight separation of the apical and basolateral regions of the epithelium. MDCK cells grown on Transwell filters polarize and form a barrier between the two chambers, so that media applied to the basal chamber does not leak components into the apical chamber [25],[26]. We added co-culture media able to support the growth of cells and *Hp* (DMEM supplemented with FBS and Brucella broth) to the basal chamber, and bathed the apical side of the cells in DMEM only, which does not support *Hp* growth (Figure 2A). Free-swimming *Hp* (∼10^8^ bacteria/ml) were added to the apical side of the polarized epithelium for 5 minutes (MOI 100∶1), and non-adherent bacteria removed by extensive washing, resulting in ∼0.1% of the inoculum attaching to the cell surface (∼1 bacterium/10 cells). Bacterial counts from the population attached to the epithelium (cell-associated) increased in accord with a *Hp* doubling time of 4–6 hours over the first day (Figure 2B). The apical surface was washed and fresh media replaced daily. Under these conditions, the cell-associated *Hp* population maintained a steady-state level of ∼10^7^ bacteria/monolayer (Figure 2B). This shows that *Hp* is able to grow and colonize the apical surface of a polarized epithelium, despite the lack of appropriate nutrients in the medium.

Simultaneously, we monitored the counts from the free-swimming (planktonic) *Hp* population, and found that the counts mirrored those of the cell-associated population, increasing over the first day, and recovering to a steady level of ∼10^6^ bacteria/chamber each day, despite washing of the epithelium and replacement of the apical media daily (Figure 2B and 2C). To determine if *Hp* grows both in the planktonic and cell-associated form on the polarized epithelium, we asked if the planktonic population grows independently of the cell-associated population. Moving the planktonic population with the apical media into a new culture well without host cells induced a rapid decrease in bacterial counts, indicating that the planktonic population does not survive independently of the host cells and the cell-associated *Hp* population (Figure 2C).

To distinguish whether *Hp* growth on the apical cell surface is due to damage of the epithelial barrier with a resultant leak of nutrients into the apical chamber, or if it instead occurs without gross disruption of monolayer integrity, we used the bacteria as sensors to test whether the apical medium becomes enriched in nutrients sufficient to support *Hp* growth. Media from the apical chambers of polarized monolayers (infected or uninfected) were collected and filtered to remove bacteria, before re-inoculating with fresh *Hp* grown on agar plates. Such “conditioned-apical” media from monolayers, whether infected or not, did not support growth of plate-grown *Hp* (Figure 2D). This said, the death rate of *Hp* in this conditioned-apical media was slower than in DMEM, indicating that the presence of cells does change the media composition, but not sufficiently to enable replication of planktonic bacteria. In agreement with these results, two different tests for macromolecule diffusion across the monolayers (tracking biotinylated albumin or 10 kD dextran) revealed no detectable gross leakage of macromolecules over 5 days of *Hp* infection (Figure S1 and Protocol S1).

**Figure 3:**
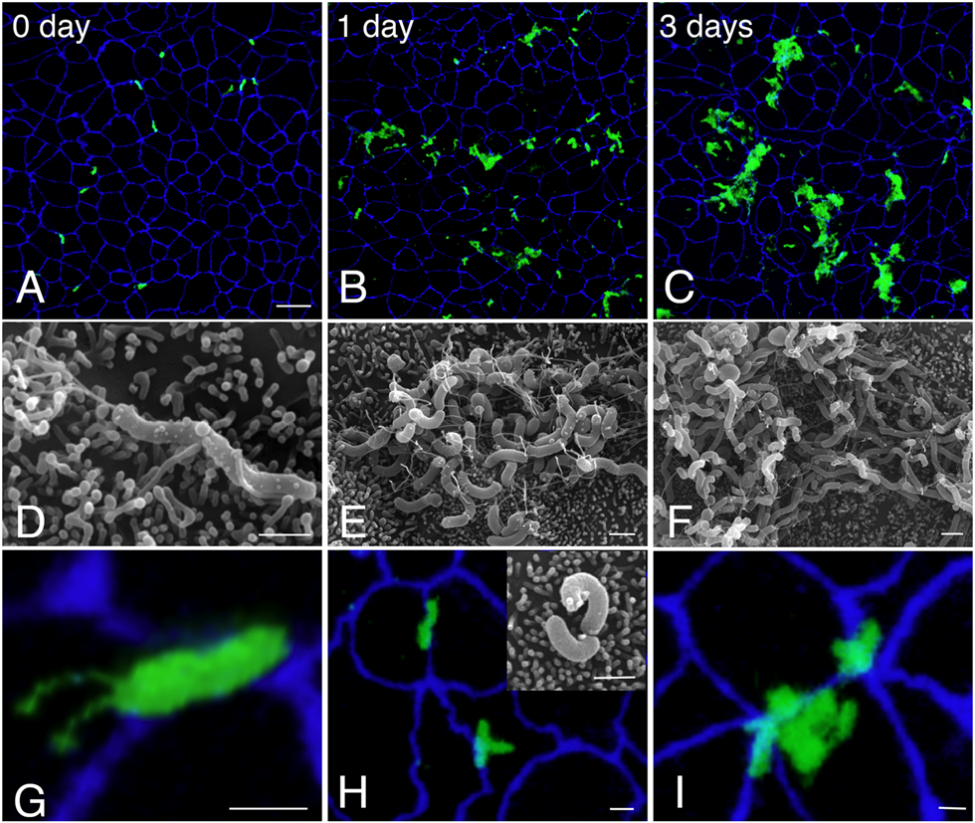
*Hp* form microcolonies on the cell surface in nutrient-poor conditions. (A–C) Low magnification 3D confocal images of WT colonizing the cell surface of polarized MDCK cells in the Transwell system. Cells were infected for 5 minutes and then unattached *Hp* washed away and media replaced (0 day). Bacteria are visualized with anti-*Hp* antibodies (green) and cell junctions are stained blue (anti-ZO-1). Scale bar 10 µm. (D–F) Scanning electron microscopy (SEM) images of WT colonizing the cell surface of polarized MDCK cells, infected as above. Scale bars 1 µm. (G–I) High magnification 3D confocal images from Transwell system infections as above, showing relation of adhered WT (green) to epithelial junctions (blue). Inset in (H) shows replicating WT as visualized by SEM. Scale bars 1 µm.

Examination of a time-course of infected monolayers on Transwell filters by confocal immunofluorescence microscopy and scanning electron microscopy (SEM) confirmed that *Hp* replicate while adhered to the polarized apical cell surface without detectable disruption of the epithelium, and despite the lack of essential nutrients in the surrounding medium. Under our experimental conditions, most of the initial attached *Hp* are single, spiral shaped, and attach directly over the apical junctions (83%) (Figure 3A, 3D, and 3G). Attachment to cellular microvilli was the most prominent initial interaction between *Hp* and the cell surface (Figure 3D). Growth of single bacteria into microcolonies was observed within 1 day after infection, and spiral bacteria in the process of dividing were noted by SEM (Figure 3B, 3E, and 3H). After 3 days of growth, larger microcolonies consisting of mostly spiral bacteria were seen (Figure 3C, 3F, and 3I).

### CagA provides a critical advantage for colonization of the apical cell surface

Having established that wild type (WT) *Hp* is able to colonize a polarized cell surface, we next examined how the *Hp* virulence factor CagA, a contact-dependent effector protein, affects bacterial survival in this niche. While much work has been done describing the effects of CagA on epithelial cells, little is known about how CagA benefits the bacteria.

We utilized a two-chambered coverglass system to simultaneously observe and compare, by time-lapse microscopy, WT and CagA-deficient mutants (Δ*cagA*) after they adhered to the cell surface. Both WT and Δ*cagA* were able to replicate on the cell surface and form microcolonies, with similar replication rates (Figure 1F). However, unlike in the Transwell system, during live-cell imaging the apical and basal compartments are not separated, the cells are not polarized, and the medium contains all the nutrients required for replication.

**Figure 4:**
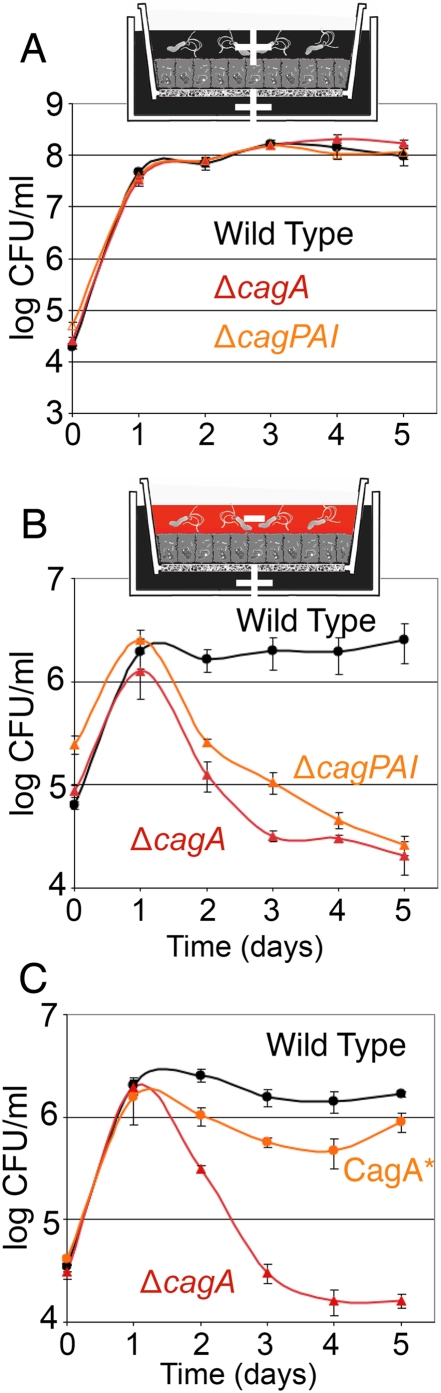
CagA is critical for *Hp* colonization of the apical cell surface. (A) Δ*cagA*, Δ*cagPAI*, and WT grow equally well in the presence of nutrients. Polarized cells on Transwell filters were infected with WT, Δ*cagA* or Δ*cagPAI*. In these experiments, co-culture media (+) was added both apically and basally. Samples were taken from the apical chamber before wash daily, and plated for CFU counts. (B,C) CagA is important in enabling *Hp* colonization of the apical cell surface. Using the Transwell system, cells were infected with strains indicated, and co-culture media added only to the basal chamber (+). DMEM was added to the apical chamber (−). Samples were taken and plated as in (A). CagA* was reconstituted from Δ*cagA*, produces an equivalent amount of CagA protein as WT, and delivers CagA to host cells (see Figure S3).

Since several reports have shown that CagA has the ability to disrupt host cell junction function and cellular polarity [17]–[21], we hypothesized that CagA might serve to provide *Hp* access to nutrients normally found only on the basolateral side of the epithelium, thereby enabling successful colonization of the apical surface. To test this hypothesis, we used the Transwell system to infect polarized MDCK monolayers from the apical side with either WT or Δ*cagA*. We also tested an isogenic *cag* pathogenicity island deletion mutant (Δ*cagPAI*). In striking contrast to WT, growth of Δ*cagA* and Δ*cagPAI* were reduced 100× (Figure 4B). Both mutant strains grow as well as WT if co-culture media is provided in the apical chamber (Figure 4A), indicating that this phenotype is not due to an *in vitro* growth defect of the mutants. Delivery of CagA into the host cells is required for effective colonization of the apical cell surface, as deletion of a TFSS gene (*cagE*) essential for CagA translocation also led to ∼100× reduction in the bacterial counts (Figure S2A). This attenuation in growth was not due to the presence of an antibiotic resistance cassette in the mutants, since an isogenic deletion mutant in the *ureA* and *ureB* genes (Δ*ureAB*), carrying the same resistance cassette as Δ*cagA*, grew as well as WT in polarized conditions (Figure S2B). We also tested a second *Hp* strain, 7.13, that has been characterized for its ability to deliver CagA *in vitro* and *in vivo*, and which causes gastric cancer in a Mongolian gerbil model of infection [27],[28]. As with the G27-MA strain, 7.13 also exhibits a defect in colonization of the apical cell surface when the *cagA* gene is deleted (Figure S2C), indicating that this phenotype is not strain specific.

Finally, we complemented Δ*cagA* with the *cagA* gene. The reconstituted strain (CagA*) produces equivalent amounts of CagA protein as WT, delivers CagA to epithelial cells (Figure S3 and Protocol S1), and recovers the ability to colonize the apical cell surface of a polarized epithelium (Figure 4C).

**Figure 5:**
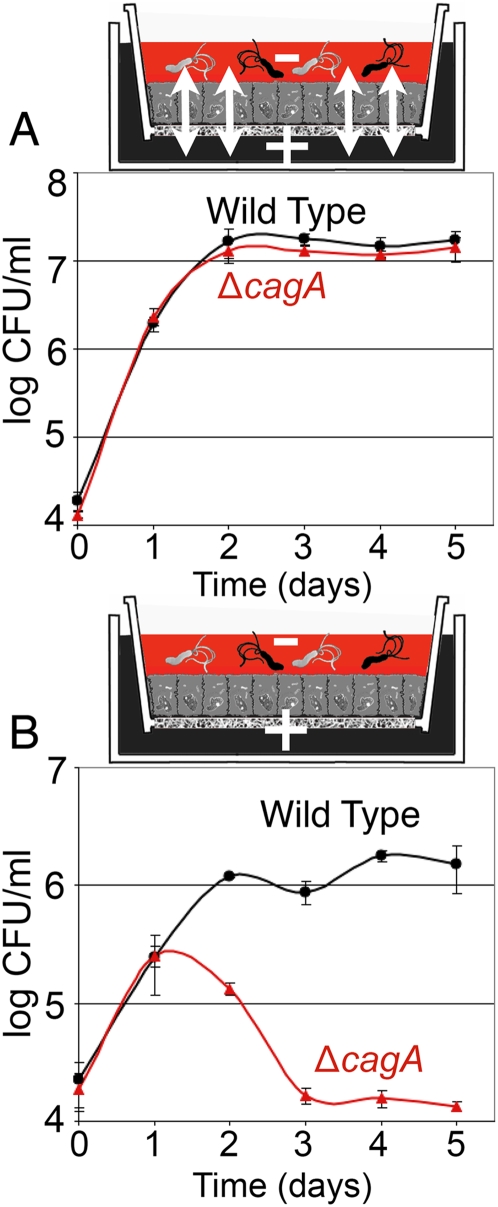
Effects of CagA are highly localized. (A) In the presence of nutrients, WT and Δ*cagA* grow equally well in a mixed infection. WT and Δ*cagA* were mixed together, and the mixture used to infect a monolayer on a Transwell filter previously fixed to induce leakiness (see Figure S1C). Samples were taken from the apical chamber before wash daily and plated on both non-selective and selective plates to differentiate WT and Δ*cagA* for CFU counts. (B) WT does not rescue Δ*cagA* in a mixed infection on an intact, polarized monolayer. The same mixture of WT and Δ*cagA* as in (A) was allowed to infect an intact polarized monolayer, and samples from the apical chamber plated daily as in (A).

To determine if CagA acts globally on the epithelium or locally at the site of individual microcolony growth, we tested whether WT would rescue Δ*cagA* if both co-infected the same monolayer. We first determined that a 1∶1 mixture of WT and Δ*cagA* in the apical chamber have similar growth kinetics in the presence of nutrients by inoculating a fixed monolayer. Fixation makes the monolayers permeable to nutrients and macromolecules from the basal chamber (Figure S1D). Mixed WT and Δ*cagA* grow equally well under these conditions (Figure 5A). In contrast, when we infect live, polarized monolayers, WT are able to survive and grow, but do not rescue the growth of Δ*cagA* (Figure 5B). Similar results were obtained with a mixture of 7.13 WT and 7.13 Δ*cagA* (Figure S2D). These results indicate that CagA's role in enhancing colonization of the cell surface is highly localized.

**Figure 6:**
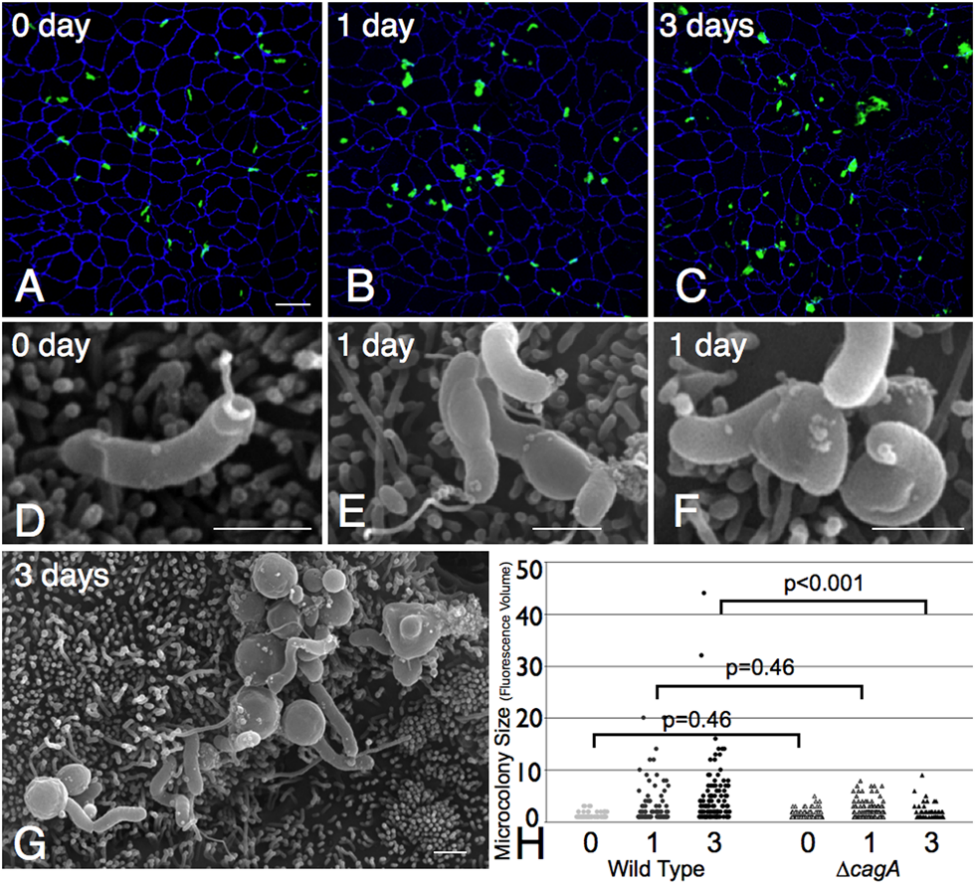
Δ*cagA* form aborted microcolonies on the apical cell surface. (A–C) Low magnification 3D confocal images of Δ*cagA* colonizing the cell surface of polarized MDCK cells in the Transwell system, as done for WT in Figure 3. Cells were infected for 5 minutes and then unattached Δ*cagA* washed away and media replaced (0 day). Bacteria are visualized with anti-*Hp* antibodies (green) and cell junctions are stained blue (anti-ZO-1). Scale bar 10 µm. (D–G) SEM images of Δ*cagA* colonizing the cell surface of polarized MDCK cells, infected as above. Scale bars 1 µm. (H) Quantitative data of WT (circles) and Δ*cagA* (triangles) microcolony sizes over time (0, 1 and 3 days), determined by fluorescence volume measured with Volocity software from multiple 3D confocal images. Each point on the graph represents a microcolony. P-values were obtained with a Mann-Whitney statistical test.

To examine how lack of CagA affects the growth of microcolonies on the cell surface, we used confocal immunofluorescence microscopy and SEM to examine a time-course of polarized cells infected with Δ*cagA*. Initial adherence of Δ*cagA* to the apical cell surface (14 bacteria/100 cells) was not lower than WT (9 bacteria/100 cells) (Figure 6A). After 5 minutes of infection, both adhered preferentially to intercellular junctions (83% of WT vs. 74% of Δ*cagA*, p = 0.2), and were spiral shaped (Figure 6D). However, Δ*cagA* exhibit a defect in microcolony formation on the cell surface, forming significantly smaller microcolonies than WT by day 3 post-infection (Figure 3C and 3F versus Figure 6C, 6G, and 6H). In addition, there were striking differences present at the ultrastructural level even by day 1 post-infection, with almost half of Δ*cagA* in various stages of coccoid formation, exhibiting swelling of the cell body and degenerate flagella (Figure 6E and 6F). At later time points (3 days post-infection), Δ*cagA* consisted mostly of aggregates of coccoid bacteria (Figure 6G). The morphology of the free-swimming bacteria in the apical chamber supernatant was also different in WT vs. Δ*cagA* beginning after one day of infection (Figure S4 and Protocol S1). Both strains initially consisted of spiral bacteria with polar flagella, but over time, the apical supernatant from the Δ*cagA* infection had increasing numbers of coccoid bacteria, and the viable counts decreased (Figure S4). Together, these data show that CagA action serves as a mechanism by which *Hp* is able to successfully colonize the apical surface of a polarized epithelium.

### CagA disruption of epithelial polarity is important in *Hp* colonization of the cell surface

CagA has the ability to affect both barrier function of an epithelium, as well as to disrupt cell polarity [17]–[21]. We asked whether CagA is providing localized leakage of nutrients from the interstitial space or whether CagA affects the cells themselves to provide *Hp* with the nutrients required for growth. As shown above, in our experimental conditions, intercellular leakage appears to be minimal, since WT does not rescue Δ*cagA* in a mixed infection on a polarized epithelium, and we do not observe significant leakage of solutes across the monolayer. We thus asked whether WT is still able to colonize and grow on the cell surface of a polarized epithelium in the absence of extracellular nutrients provided in the basal media.

**Figure 7:**
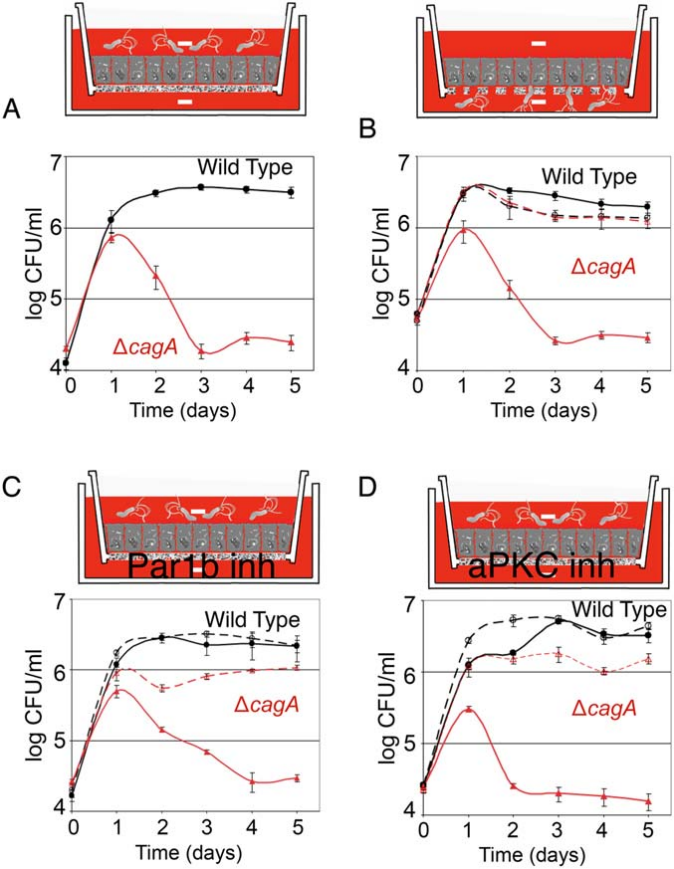
Polarity disruption is involved in *Hp* colonization of the apical cell surface. (A) *Hp* can acquire nutrients for growth directly from the epithelium. Polarized cells on Transwell filters were infected with WT or Δ*cagA* apically, in the presence of plain DMEM in both apical and basal chambers. Samples were taken from the apical chamber before wash daily, and plated for CFU counts. (B) The apical vs. basolateral cell surfaces are intrinsically different in their ability to support *Hp* growth. Polarized cells on Transwell filters with larger 3 µm pores were infected with WT or Δ*cagA* either from the apical (solid lines) or basal side (dashed lines), in the presence of plain DMEM in both chambers. Samples from the appropriate chamber were taken before wash daily and plated for CFU counts. (C,D) Inhibition of atypical protein kinase C aPKC or Par1b rescues Δ*cagA* growth. Polarized monolayers were infected apically as in (A). Solid lines indicate conditions with plain DMEM in both apical and basal chambers. Dashed lines indicate conditions with 20 µM hymenialdisine, a Par1b inhibitor, added to DMEM in the basal chamber (panel C), or conditions with a specific aPKCζ pseudosubstrate inhibitor (10 µM) added to DMEM in the basal chamber (panel D).

We generated polarized cells and then replaced both apical and basal media with DMEM at the time of infection. We found that WT is still able to survive and grow on the apical cell surface, while Δ*cagA* exhibits the same ∼100× defect in colonization observed previously (Figure 7A). This result implies that *Hp* is able to obtain needed nutrients from the epithelial cells themselves. It also suggests the hypothesis that epithelial polarity, independently of the barrier function of the junctions, is an important target of pathogenesis, and that CagA's effects on cell polarity may be involved in colonizing the apical cell surface.

To begin testing this hypothesis, we asked whether the basolateral vs. apical surfaces of the epithelium have intrinsically different abilities to support the growth of *Hp*. We repeated the Transwell system experiment above, but infected the cells either from the apical or basal side, and utilized filters with larger 3 µm pores to allow *Hp* access to the basolateral surface of the cells. Strikingly, when the bacteria are allowed to infect from the basal side, where they have access to the basolateral surface of the polarized epithelial cells, Δ*cagA* grow as well as WT (Figure 7B).

The results above suggest that cell polarity is a deterrent to *Hp* colonization of the cell surface, and that CagA functions in colonization of this niche by perturbing cell polarity. This implies that disrupting cell polarity should result in rescue of Δ*cagA*, allowing it to now colonize the apical cell surface. To test this, we targeted the atypical protein kinase C (aPKC)/Par1b pathway which plays an important role in the establishment and maintenance of cell polarity [29]–[31], and is known to be disrupted by CagA [19],[21]. Addition of a Par1b inhibitor, hymenialdisine [32],[33], led to rescue of Δ*cagA*'s defect in colonizing the apical cell surface, while not affecting WT growth (Figure 7C). Similarly, inhibition of aPKCζ with a specific pseudosubstrate inhibitor [31] also rescued Δ*cagA* (Figure 7D). Together, these results show that CagA perturbation of host cell polarity is a key mediator of the ability of *Hp* to colonize the apical cell surface of a polarized epithelium.

## Discussion

While many microbial infections disrupt the epithelial barrier in order to cause damage, reach deeper tissues or disseminate, here we present the concept that, in addition, some pathogenic bacteria affect epithelial polarity to use the cell surface as a replicative niche. We found that *Hp* is able to replicate while remaining adhered to the cell surface, forming clonal microcolonies. Microcolony formation on the cell surface has been observed for *Hp* in human infection, as well as in several other mucosal colonizers [2]–[6]. Our observations suggest that *Hp* and other microbes may be taking advantage of the cell surface in order to exploit a niche that is distinct from growth in epithelial secretions.

We were able to begin dissecting whether cell-associated *Hp* can grow independently of the planktonic population by mimicking intact tissue polarity using a Transwell filter system. Epithelial cell polarity is the ability to generate distinct apical vs. basolateral surfaces, as well as the maintenance of a barrier between the lumenal vs. interstitial spaces. This has been studied mostly in the context of cellular development and physiology in which tissue maintenance, wound healing and vectorial transport of nutrients and ions are controlled by epithelial polarity [24],[34]. However, most epithelia also serve as a barrier to microbial invasion, and are constantly exposed through their apical side to interactions with microbes. As such, some aspects of epithelial polarity may have also evolved to deter bacterial colonization of the apical cell surface. We found that *Hp* survive, grow, and form microcolonies on the apical surface of a polarized monolayer, in conditions where the free-swimming *Hp* population is rapidly killed.

To examine the molecular mechanisms involved in cell surface colonization, we tested the role of the cag TFSS and CagA. CagA is the only known effector protein translocated into host cells through *Hp*'s TFSS. CagA-positive strains are correlated with more severe disease outcome [35]–[37], and CagA's effects on the host cell have been a topic of intense study. In addition to its effects on cellular polarity and tight junction function [17]–[21], CagA is also known to activate receptor tyrosine kinase growth factor-like signaling [38]–[41] and affect cellular turnover [42]. These effects of CagA may be important in its ability to act as a bacterial oncogene, leading to the development of carcinoma [43]. However, the fundamental question of how CagA might act to benefit *Hp* has remained unclear, although a recent report showed that *Hp* Δ*cagA* mutants are less effective in colonizing the stomach in a Mongolian gerbil model of *Hp* infection [42].

We found that Δ*cagA* mutants became coccoid in morphology as early as one day after infection of a polarized epithelium, and formed small aborted microcolonies on the cell surface. A coccoid morphology in *Hp* is associated with stress conditions and thought to be a specialized form of cell death or degeneration, although there remains controversy regarding whether coccoid *Hp* are viable but non-culturable [44]–[47]. Δ*cagA* were not rescued by WT *Hp* in mixed infections, indicating the highly localized nature of CagA action in mediating the ability of *Hp* to colonize the polarized epithelium.

CagA's ability to disrupt cellular polarity and tight junction function has led to the idea that disruption of the epithelial barrier is a means to acquire nutrients located in the interstitium [48],[49]. *In vivo*, both polarity and barrier disruption may occur, since *Hp* utilize multiple strategies to perturb the epithelium [50],[51]. Our use of a simplified experimental system allows us to distinguish between CagA's effects on cell polarity vs. its effects on epithelial barrier function on *Hp* colonization of the apical cell surface. We found that CagA's effects on cell polarity, independent of its ability to disrupt barrier function, plays a key role in enabling *Hp* to turn the apical cell surface into a replicative niche. In contrast to apical infection, the Δ*cagA* mutant survived and grew as well as WT upon infection from the basolateral surface, indicating that the apical vs. basolateral membranes are intrinsically different in their ability to support *Hp* growth. Further, inhibition of the aPKC/Par1b polarity pathway, known to be affected by CagA [19],[21], led to rescue of Δ*cagA*, which was now able to survive and grow on the apical cell surface.

Our findings show that the sophisticated manipulation of cell polarity by injection of CagA is one mechanism that enables *Hp* to exploit the apical cell surface as a replicative niche. Further studies will reveal which specific nutrients *Hp* extracts from the epithelium, and how perturbations of polarity result in altering the epithelial surface for the benefit of colonizing microbes. As an example, iron is a key nutrient important for *Hp* growth that is known to be sequestered by the host [52],[53]. It has been reported that *Hp* is able to survive despite iron chelation in the medium, when grown in the presence of host cells, suggesting that the bacteria are able to extract needed iron directly from the host cells [54]. Of note, the presence of lactoferrin in mucosal secretions, where it sequesters iron, and the delivery of iron to cells via transferrin, which binds iron in the interstitium, requires their proper polarized secretion and transport [55]–[59]. We speculate that perturbation of the ability of polarized epithelial cells to vectorially direct and compartmentalize micronutrients, sequester receptors for adhesion away from microbes, or control secretion of anti-microbial factors, may all be targets for microbes that utilize the cell surface as a replicative niche.

It is also interesting to note that the delivery of CagA itself may be regulated by cell polarity, since a recent report proposed that CagL on the TFSS must interact with integrin α5β1 receptors before CagA is delivered into host cells [60]. Integrins are exclusively localized at basolateral membranes in an intact polarized epithelium [61], suggesting that the localization of initial attachment, and perhaps other means of initially perturbing polarity, are important in injecting CagA.

In the same way that intracellular pathogens have evolved diverse molecular adaptations to colonize the inside of the host cell, we suggest that the apical cell surface presents unique challenges for microbial colonization. We propose the concept that *Hp* locally modulates epithelial polarity through CagA as one strategy to colonize the cell surface. This concept may be pertinent not only for *Hp*, but also for other mucosal colonizers such as enteropathogenic *Escherichia coli*, for which microcolonies on the host cell surface have been observed [4],[5], and which is known to disrupt host cell polarity at the site of bacterial attachment [62]. *Pseudomonas aeruginosa* has also been shown to locally disrupt cell polarity [63], raising the possibility that several microbial pathogens have evolved strategies to usurp this epithelial function for their benefit. Further study of this niche is likely to reveal additional molecular mechanisms for bacterial survival, ranging from specialized adhesion, to mechanisms for microcolony formation and dispersal, and to novel strategies for perturbing the epithelial barrier and cell polarity. It also presents a novel niche of host-bacterial interactions for which therapeutic or preventive targets may be developed.

## Materials and Methods

### Cell culture

Madin-Darby Canine Kidney II (MDCK) and RFP-E-cadherin MDCK II cells (kindly provided by W. James Nelson, Stanford University, Stanford, CA) [64] were maintained in DMEM (Gibco) containing 5% fetal bovine serum (FBS) (Gibco), at 37°C in a 5% CO_2_ atmosphere. AGS and Caco-2 cells were maintained in DMEM containing 5% or 10% FBS respectively, at 37°C in a 5% CO_2_ atmosphere. Polarized MDCK monolayers were cultured by seeding cells at confluent density onto 12 mm, 0.4 µm-pore polycarbonate tissue culture inserts (Transwell filters; Corning Costar). Apical media was changed to DMEM one day after seeding. Basal media (DMEM+5% FBS) was changed daily and confluent monolayers were allowed to fully polarize for four days before use in assays. Primary murine gastric epithelial cells were isolated from three-day-old postnatal C57BL6/J mice, and maintained with a protocol from Fujikawa et al [22] and Ootani et al [23]. Animal experiments were carried out in accordance with protocols reviewed and approved by the institutional animal care and use committee of Stanford University.

### *Hp* strains and culture

*Hp* strain G27-MA has been previously described [18]. An isogenic Δ*cagPAI* mutant of G27-MA was generated using a previously described construct, kindly provided by Stefano Censini (Norvatis Vaccines, Sienna, Italy) [65]. Isogenic G27-MA Δ*cagE* and Δ*ureAB* mutants were generated using previously described constructs [18],[66]. G27-MA expressing GFP was generated by transformation with plasmid pTM115, containing the enhanced green fluorescent protein under the control of the urease promoter [18]. *Hp* strain 7.13 was kindly provided by Richard Peek (Vanderbilt University, Nashville, TN) and has been previously described [27]. Isogenic Δ*cagA* mutants of strains G27-MA and 7.13 were constructed by deletion of the complete open reading frame (ORF) of *cagA* and replacement with the *cat* gene (conferring chloramphenicol resistance), by a PCR based method without recombinant cloning [66],[67]. Complementation of G27-MA Δ*cagA* was achieved by natural transformation with a construct containing the *cagA* ORF with the *aphA* gene (conferring kanamycin resistance) immediately downstream, flanked by the upstream and downstream regions of *cagA* to allow for homologous recombination. Routine culture of *Hp* on Columbia blood agar plates and co-culture of *Hp* with MDCK cells were as previously described [18],[68]. Unless otherwise indicated, *Hp* from co-cultures were used for infections. Co-culture media for *Hp* with MDCK cells consists of DMEM+5% FBS+10% Brucella broth+10 µg/ml vancomycin. For *Hp* growth experiments in media without cells, *Hp* grown overnight on Columbia blood agar plates were resuspended in DMEM, and aliquots inoculated into the appropriate media in 6-well plates.

### *Hp* infection of epithelial cells on glass coverslips

MDCK, AGS, or Caco-2 cells were grown on glass coverslips in 6-well plates. *Hp* were allowed to adhere for 5 minutes and then cells washed five times with fresh DMEM to remove non-adherent bacteria. Co-culture media was added back to the wells, and incubated at 37°C in a 5% CO_2_ atmosphere. Before harvesting, samples were washed 3 times with fresh DMEM, and the coverslips fixed for 10 minutes with 2% paraformaldehyde in 100 mM phosphate buffer (pH 7.4).

### Live-cell time-lapse microscopy

Cells were seeded on 35 mm glass bottom microwell dishes (MatTek) or on Lab-TeK II two-chambered coverglass (Nalge Nunc International) for simultaneous imaging of two conditions. *Hp* were allowed to adhere for 1 minute, and the cells washed three times with DMEM to remove non-adhered bacteria. Cells were then washed an additional two times with co-culture media without phenol red. Imaging was performed using a Nikon TE2000E microscope equipped with high numerical aperture objectives for imaging at low light levels to minimize phototoxicity, and a stage enclosed in a complete Solent incubator system with temperature and CO_2_ enrichment controls. A motorized, computer-controlled stage was used to sequentially monitor multiple sites of the same specimen over time in both transmitted light (DIC) and multi-channel fluorescence. For DIC imaging, a Hamamatsu high resolution ORCA-285 digital camera was used. For fluorescence imaging, a high gain, high speed, low noise, Hamamatsu Electron Multiplier C9100-12 back-thinned CCD camera was used. A z-stack of images was collected at each time point and processed with OpenLab software (Improvision).

### Transwell *Hp* growth assays

Just prior to infection, fresh co-culture media was added to the basal chamber. For experiments in DMEM alone, the basal chamber was washed 5 times with fresh DMEM before final addition of DMEM. *Hp* (∼10^8^ bacteria/ml) were added to the apical chamber, allowed to adhere for 5 minutes, and cell monolayers then washed 5 times with fresh DMEM to remove non-adherent bacteria. Appropriate media was added back to the apical chamber, and the cells incubated at 37°C in a 5% CO_2_ atmosphere. Basal media was changed daily. After sampling for colony-forming unit (CFU) counts from the apical chamber each day, cell monolayers were washed 3 times with fresh DMEM, before appropriate media added back and the cells returned to the incubator. For mixed infections, samples were also plated on Columbia blood agar plates containing 25 µg/ml chloramphenicol, in order to differentiate the two strains. Data are shown as means±SD. For counts of cell-associated *Hp*, after the cell monolayers were washed 3 times with fresh DMEM, they were incubated for 5 minutes with DMEM containing 1% saponin (Sigma), and the monolayers then scrapped off the filter. The cells were dispersed by 8 passages through a 27-gauge syringe, and samples plated for CFU counts. Where used, the Par1b inhibitor hymenialdisine (Biomol International) was added at a final concentration of 20 µM, and the myristoylated PKCζ pseudosubstrate inhibitor (United States Biological) was added at a final concentration of 10 µM to the media in the basal chamber. For assays with pre-fixed cells, cell monolayers were fixed for 10 minutes with 2% paraformaldehyde in 100 mM phosphate buffer (pH 7.4), and then washed 5 times with PBS before equilibrating with media and *Hp* infection.

Experiments of apical vs. basal surface infection were carried out with polarized MDCK cells on 3 µm-pore polycarbonate tissue culture inserts (Transwell filters; Corning Costar). *Hp* (∼10^8^ bacteria/ml) were added either to the apical or basal chamber, and allowed to adhere for 5 minutes, before washing with DMEM 5 times to remove non-adherent bacteria. DMEM was added to both chambers, and the cells incubated. CFU counts were sampled from the appropriate chamber each day before wash and media replacement.

### Conditioned-apical media assay

Media from the apical chamber from uninfected or *Hp* infected monolayers on Transwells were collected 1 day after infection, and filtered through a 0.22 µm pore to remove all bacteria (“conditioned-apical media”). *Hp* grown overnight on Columbia blood agar plates were resuspended in DMEM, used to inoculate this conditioned-apical media in a 96-well plate, and the plate incubated at 37°C in a 5% CO_2_ atmosphere. Samples were taken at several time points and dilutions plated for CFU counts. Data are shown as means±SD.

### Confocal immunofluorescence microscopy and antibodies

Samples were processed for confocal immunofluorescence as previously described [26]. Mouse anti-ZO-1 antibodies (Zymed) were used at 1∶300 dilution. Chicken anti-*Hp* antibodies [18] were used at 1∶200 dilution. Anti-IgG Alexa-fluor conjugated antibodies of appropriate fluorescence and species reactivity (Molecular Probes) were used for secondary detection. Alexa-fluor 647 phalloidin (Molecular Probes) was used for visualization of the actin cytoskeleton. Samples were imaged with a BioRad Confocal MRC-1024 microscope, and z-stacks reconstructed into 3D using Volocity software (Improvision). For quantification of microcolony sizes, we randomly sampled 100 µm×100 µm optical fields by confocal microscopy. The 3D reconstructions of the confocal stacks were used to collect the fluorescence voxel volume of each microcolony stained with anti-*Hp* antibodies. Microcolony fluorescence volumes from WT and Δ*cagA* infected monolayers were compared by non-parametric Mann-Whitney test.

### Scanning electron microscopy

Samples were fixed with 4% Paraformaldehyde and 2% Glutaraldehyde in 0.1 M Sodium Cacodylate Buffer (pH 7.2) and post-fixed with 1% OsO_4_ in water. Samples were dehydrated in an ascending ethanol series before critical point drying with liquid CO_2_ in a Tousimis AutoSamdri-814 apparatus. Transwell filters were mounted on adhesive carbon films on 12 mm aluminum stubs (Ted Pella), and sputter-coated with 100 Å of Au/Pd using a Denton Desk 11 Sputter Coater. Visualization was performed with a Hitachi S-3400N Variable Pressure SEM operated at 15 kV, working distance 7–8 mm, and secondary electron detector under high-vacuum conditions (<1 Pa). Images were captured in TIFF format.

## Supporting Information

Protocol S1 Supplementary Materials and Methods.
(0.06 MB PDF)

Figure S1 Tracking of solute diffusion across polarized monolayers. (A) Determination of detection limit and linear range of biotin-albumin by western blot. Biotin-albumin from a 2 mg/ml stock solution dissolved in PBS was diluted into DMEM starting at 1 µg/ml. These were further diluted 1∶1 in SDS-sample buffer, boiled, and an amount of biotin-albumin equivalent to 0.2 ng to 10 ng was loaded and separated by SDS-PAGE and transferred to a nitrocellulose membrane. The membrane was probed with Alexa-fluor 647-conjugated streptavidin and bands visualized by the LI-COR Odyssey Scanner. The 24-bit data scan of each band was quantified by determining the integrated intensity of the pixel signals using the Odyssey software. Background was detected by obtaining readings of the integrated intensity from 5 other areas in the blot. Arbitrary units were used for the integrated intensity graph. The data was plotted as integrated intensity minus the background. A best-fit linear curve for the data is shown, as is the linear formula and its fit. The detection limit was around 0.2 ng, which represents a concentration of 20 ng/ml of biotin-albumin in the apical chamber or 0.13% of what would be expected if there were free diffusion between the basolateral and apical chambers. (B) Five-day *Hp* infection does not lead to gross disruption of monolayer integrity. 30 µg of biotin-albumin was added to the basal chamber of uninfected or infected polarized monolayers. The apical supernatant was sampled daily and 10 µl of these samples separated by SDS-PAGE, blotted onto nitrocellulose and the biotinylated albumin visualized with fluorescent streptavidin. Each band was quantified with the Odyssey Scanner (see Figure S1A). The solid line plots the concentration of biotin-albumin detected each day in the apical chamber of the infected cells. The dotted line shows a similar plot with samples collected from uninfected Transwell chambers. In uninfected polarized monolayers we detected a daily apical concentration of 50±2 ng/ml (0.33%) constantly over 8 days. With *Hp* infected monolayers we found similar levels of apical biotin-albumin (57±2 ng/ml 0.38%) in the first 5 days of infection. By 8 days of infection a higher amount of transfer was detected in infected monolayers (80 ng/ml) but still only accounting for 0.53% of what would be expected if there were free diffusion of macromolecules between the two chambers. (C) Determination of detection limit and linear range of 10 kD Alexa-fluor 647 dextran by the LI-COR Odyssey Scanner. A dilution series starting with 100 µg/ml of fluorescent dextran in DMEM was made in a 96-well plate. All samples were in triplicate, and fluorescence directly detected using the LI-COR Odyssey Scanner. Scan data was obtained and a best-fit linear curve determined as in (A). The detection limit was around 1.5 µg/ml. (D) *Hp* infection does not lead to gross leakage of basal contents into the apical chamber. Using the Transwell system, live or fixed cells were either left uninfected or infected with WT. After 1 day, fresh DMEM was added to the apical chamber, and 100 µg/ml fluorescent 10 kD Alexa-fluor 647 dextran in DMEM added to the basal chamber for 30 minutes. Samples were then taken from the apical chamber, and fluorescence directly measured in a LI-COR Odyssey Scanner. Data is presented as percent diffusion of starting amount of dextran into the apical chamber. Detection limit (2%) is indicated by the line labeled “Bkg”. Fixing the monolayers perturbs barrier function, allowing diffusion of basal solutes into the apical compartment. With fixed uninfected or infected monolayers we detected about 10–12% diffusion of dextran apically in 30 minutes. With live uninfected or infected monolayers we could not detect any transfer of 10 kD dextran into the apical chamber.
(0.15 MB PDF)

Figure S2 CagA is critical for *Hp* colonization of the apical cell surface. (A–C) Cells were infected in the Transwell system with strains indicated, and co-culture media added only to the basal chamber (+). DMEM was added to the apical chamber (−). Samples were taken from the apical chamber before wash daily, and plated for CFU counts. Δ*cagE* is a mutant defective in the ability to translocate CagA into host cells. Δ*ureAB* is deleted for the *ureA* and *ureB* genes, marked with the same chloramphenicol resistance cassette as used in Δ*cagA*. 7.13 is an unrelated *Hp* strain, previously characterized for its ability to deliver CagA [27]. (D) Δ*cagA* is not rescued by WT in a mixed infection. 7.13 WT and Δ*cagA* were mixed together, and the mixture used to infect a monolayer on a Transwell filter. A monoculture of Δ*cagA* was also used to infect a separate Transwell filter (dashed line) at the same time. Samples were taken from the apical chamber before wash daily and plated on both non-selective and selective plates to differentiate WT and Δ*cagA* for CFU counts.
(0.35 MB PDF)

Figure S3 Reconstituted CagA° delivers equivalent amounts of CagA as WT. MDCK cells were infected with the indicated strains, and the infection allowed to proceed for 24 hours. Lysates from these infections were separated by SDS-PAGE, transferred to a nitrocellulose membrane, then immunoblotted with antibodies raised against the CagA-N-terminus (green, panel C) and against phosphorylated CagA (red, panel D). Panel B shows the merge of the anti-CagA-NT and anti-phospho-CagA blots. Panel A is a Coomassie Blue-stained gel showing total protein loaded.
(0.25 MB PDF)

Figure S4 Change in morphology of free-swimming Δ*cagA* in the Transwell system. Polarized cells in the Transwell system were infected apically with WT or Δ*cagA*, and samples taken daily before wash for CFU counts (graph). At initial infection (Day 0), and at days 1 and 3 post-infection, samples were also taken from the apical chamber and the free-swimming bacteria examined by whole-mount TEM negative staining. Scale bars 1 µm.
(1.65 MB PDF)

Video S1 DIC time-lapse of *Hp* replication on MDCK cell surface. DIC time-lapse movie showing two *Hp* replicating over the intercellular junctions on a confluent monolayer of MDCK cells. Arrows track the bacteria as they replicate. Images were collected every minute, over a period of 6.75 hours. The movie is compressed into 45 seconds (540× speed).
(8.66 MB MOV)

Video S2 DIC time-lapse of *Hp* replication on gastric primary cells. DIC time-lapse movie of *Hp* replicating while adhered to the membrane of primary murine gastric epithelial cells. Images were collected every 5 minutes, over a period of 12.5 hours. The movie is compressed into 10 seconds (4500×).
(2.58 MB MOV)

Video S3 Live fluorescence time-lapse of *Hp* replication on MDCK junctions. Time-lapse fluorescence microscopy of GFP-*Hp* (green) replicating over the epithelial junctions of MDCK cells expressing RFP-E-cadherin (red). Cells were infected on the microscope stage with GFP-expressing *Hp*. Images were collected every 20 minutes, over a period of 6.9 hours. The movie is compressed into 3.6 seconds (6900×).
(0.35 MB MOV)

Video S4 Live fluorescence time-lapse of *Hp* microcolony formation. A longer time-lapse fluorescence recording of cells infected as in Video S3, starting a day after initial infection, tracking GFP-*Hp* (green) that began as a small cluster over the epithelial junctions. Images were collected every 20 minutes, over a period of 20.7 hours. The movie is compressed into 7.65 seconds (9700×).
(4.84 MB MOV)
