# Supplementary material for: Helicobacter pylori Usurps Cell Polarity to Turn the Cell Surface into a Replicative Niche
Source: PLoS Pathog. 2009 May 1;5(5):e1000407. doi: 10.1371/journal.ppat.1000407 (PMC2669173; doi:10.1371/journal.ppat.1000407)
Supplement: Protocol S1 — Supplementary Materials and Methods. (0.06 MB PDF) [file ppat.1000407.s001.pdf]

Protocol S1- Supplementary Materials and Methods

Monolayer solute leakage assays

Biotinylated albumin (Sigma) was added at 20 µg/ml to the media in the Transwell basal chamber, and replaced daily. The basal chamber contains 1.5 ml of media, while the apical chamber contains 0.5 ml of media. The media from the apical chamber was sampled daily. Detection of biotinylated albumin in the samples was carried out as described previously [18]. The detection limit and linear range of measurements of the biotinylated albumin was determined from a standard curve generated by use of a dilution series of a 1 µg/ml solution of biotinylated albumin in DMEM, diluted 1:1 in SDS-sample buffer.
For the fluorescent dextran monolayer integrity assay, fluorescent 10 kD Alexa-fluor 647 dextran (Molecular Probes) was added at a final concentration of 100 µg/ml in DMEM to the basal chamber. Fresh DMEM was added to the apical chamber. After incubation for 30 minutes, samples were taken from the apical chamber, and the fluorescence directly measured in a LI-COR Odyssey Scanner (LI-COR Biosciences). A dilution series of the 100 µg/ml fluorescent dextran solution was made and a standard curve generated to determine the detection limit and linear range of the measurements.

MDCK cell infection and CagA delivery detection

Antibodies for detection of CagA and phospho-CagA were made as follows. For detection of non-phosphorylated CagA, we expressed the first 877 amino acids of G27 CagA in *E. coli* as a GST fusion, and used this peptide to generate antibodies in rabbits (Covance). This peptide lacks the EPIYA motifs. For phospho-CagA detection, a Keyhole Limpet Hemocyanine phospho-peptide containing the sequence CZSVSPEPI[pY]ATIDD based on the G27 strain 3rd EPIYA motif was synthesized and used to generate antisera in chickens (Aves Labs Inc). Antibodies were affinity purified with the phospho-peptide and cross-adsorbed against synthetic non-phosphorylated peptide. The affinity purified antibodies were further pre-absorbed with WT G27-MA bacteria that were fixed in 2% paraformaldehyde and permeabilized with 1% saponin and 0.1% Triton X-100 to remove all antibodies cross reacting to non-phosphorylated CagA. MDCK cells were maintained in DMEM containing 5% FBS (Gibco). Cells were seeded in 6-well plates for *Hp* infections. *Hp* were allowed to attach to the cells for 5 minutes, before the cells were washed 3 times to remove non-adherent bacteria, and co-culture media added back to the wells. 24 hours after infection, cells were washed three times with DMEM, and boiling SDS-sample buffer added. Lysates were collected, boiled and spun down. Samples were separated by SDS-PAGE, and immunoblotted with antibodies raised against the CagA-N-terminus and against a phosphorylated CagA peptide. To visualize total protein, SDS-PAGE gel was stained with Coomassie Blue (Sigma). A LI-COR Odyssey Scanner was used for signal detection (LI-COR Biosciences).

Transmission electron microscopy

For negative staining, samples of *Hp* grown in continuous co-culture with MDCK cells or from the apical supernatants of infected Transwells were taken and Formvar carbon-coated grids floated on drops of the bacterial samples for 5 minutes. Grids were then washed in distilled water, and stained with 1% aqueous uranyl acetate for 2 minutes. The grids were examined and photographed in a Philips CM-12 transmission electron microscope.
