# Supplementary figures and images for: Helicobacter pylori Usurps Cell Polarity to Turn the Cell Surface into a Replicative Niche
Source: PLoS Pathog. 2009 May 1;5(5):e1000407. doi: 10.1371/journal.ppat.1000407 (PMC2669173; doi:10.1371/journal.ppat.1000407)

Figure S1

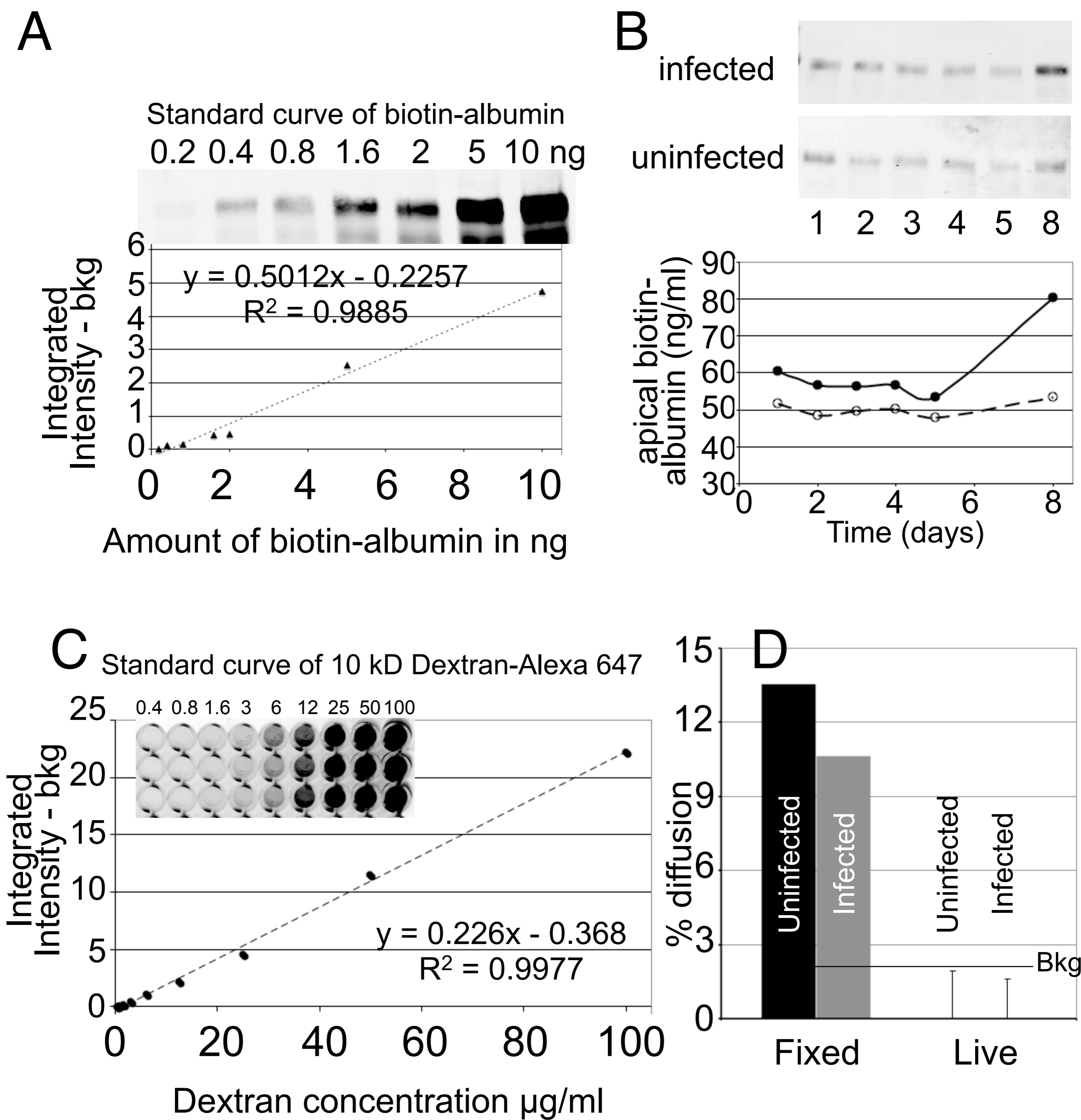

Supplement: Figure S1 — Tracking of solute diffusion across polarized monolayers. (A) Determination of detection limit and linear range of biotin-albumin by western blot. Biotin-albumin from a 2 mg/ml stock solution dissolved in PBS was diluted into DMEM starting at 1 µg/ml. These were further diluted 1∶1 in SDS-sample buffer, boiled, and an amount of biotin-albumin equivalent to 0.2 ng to 10 ng was loaded and separated by SDS-PAGE and transferred to a nitrocellulose membrane. The membrane was probed with Alexa-fluor 647-conjugated streptavidin and bands visualized by the LI-COR Odyssey Scanner. The 24-bit data scan of each band was quantified by determining the integrated intensity of the pixel signals using the Odyssey software. Background was detected by obtaining readings of the integrated intensity from 5 other areas in the blot. Arbitrary units were used for the integrated intensity graph. The data was plotted as integrated intensity minus the background. A best-fit linear curve for the data is shown, as is the linear formula and its fit. The detection limit was around 0.2 ng, which represents a concentration of 20 ng/ml of biotin-albumin in the apical chamber or 0.13% of what would be expected if there were free diffusion between the basolateral and apical chambers. (B) Five-day Hp infection does not lead to gross disruption of monolayer integrity. 30 µg of biotin-albumin was added to the basal chamber of uninfected or infected polarized monolayers. The apical supernatant was sampled daily and 10 µl of these samples separated by SDS-PAGE, blotted onto nitrocellulose and the biotinylated albumin visualized with fluorescent streptavidin. Each band was quantified with the Odyssey Scanner (see Figure S1A). The solid line plots the concentration of biotin-albumin detected each day in the apical chamber of the infected cells. The dotted line shows a similar plot with samples collected from uninfected Transwell chambers. In uninfected polarized monolayers we detected a daily apical concent [file ppat.1000407.s002.pdf]

Figure S2

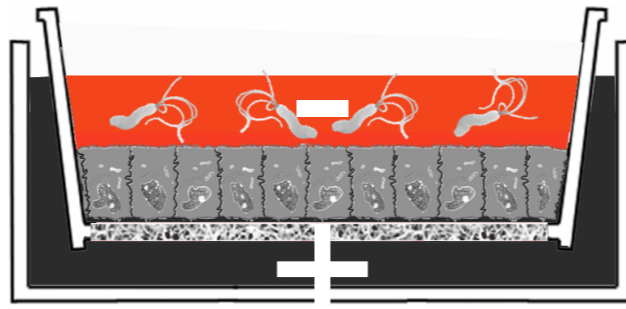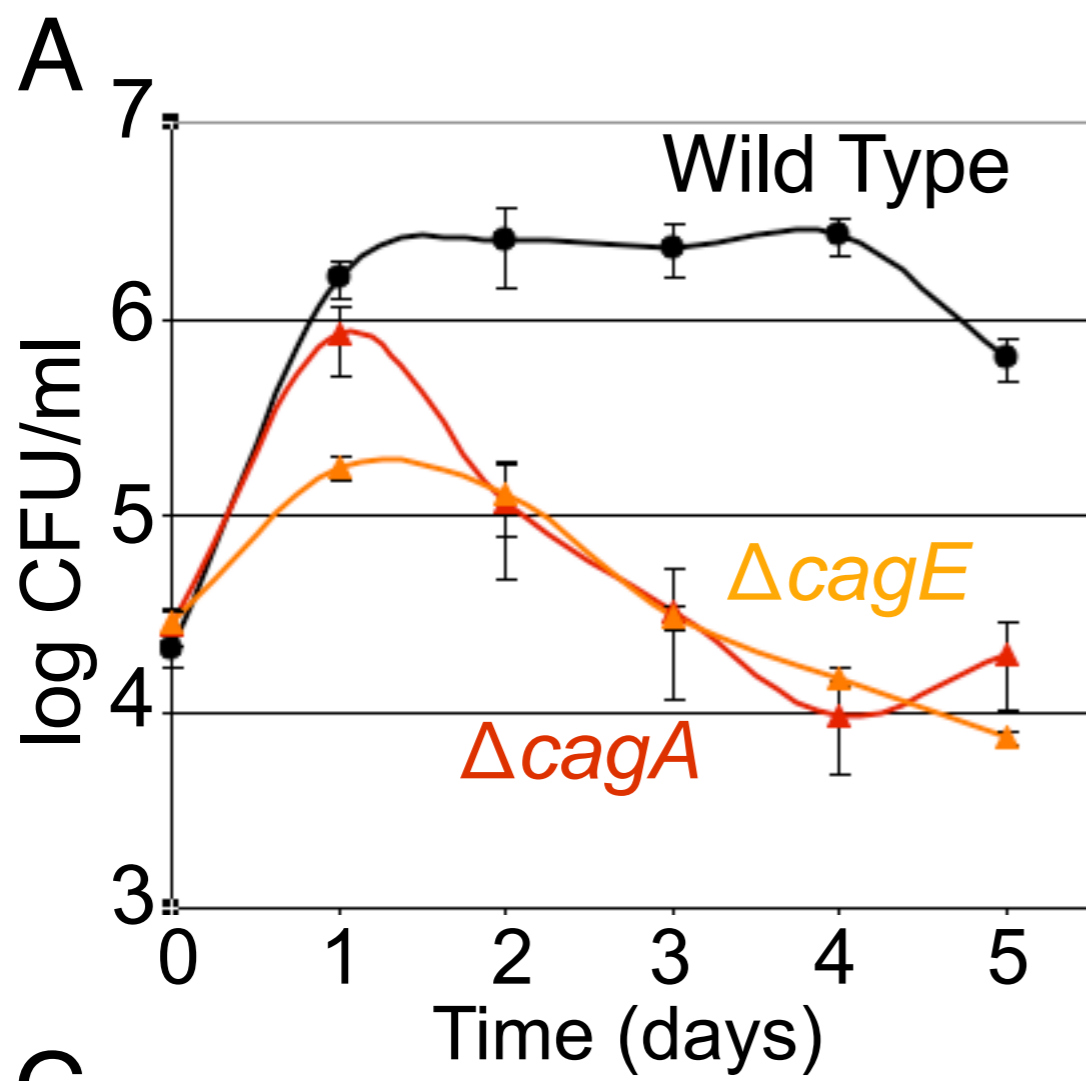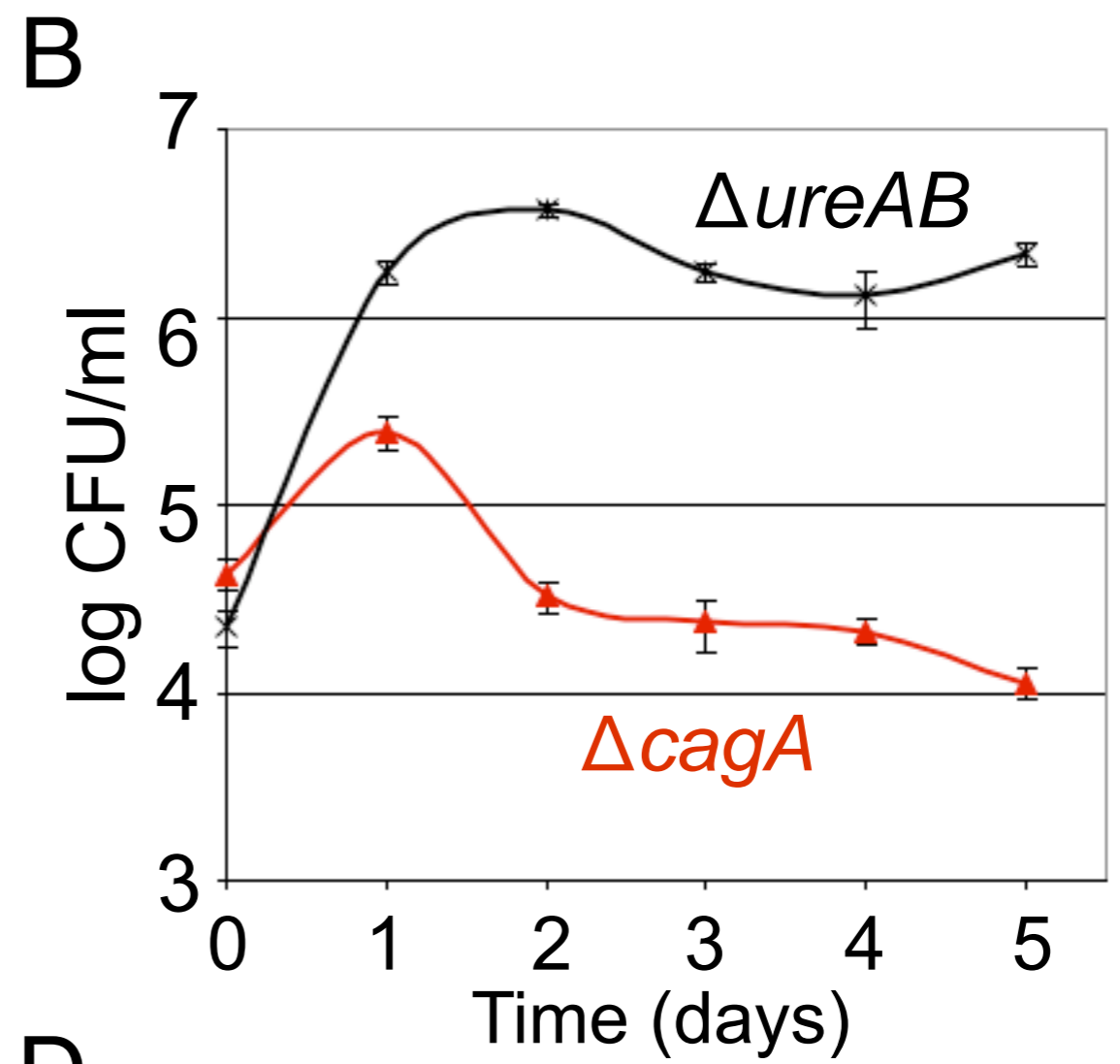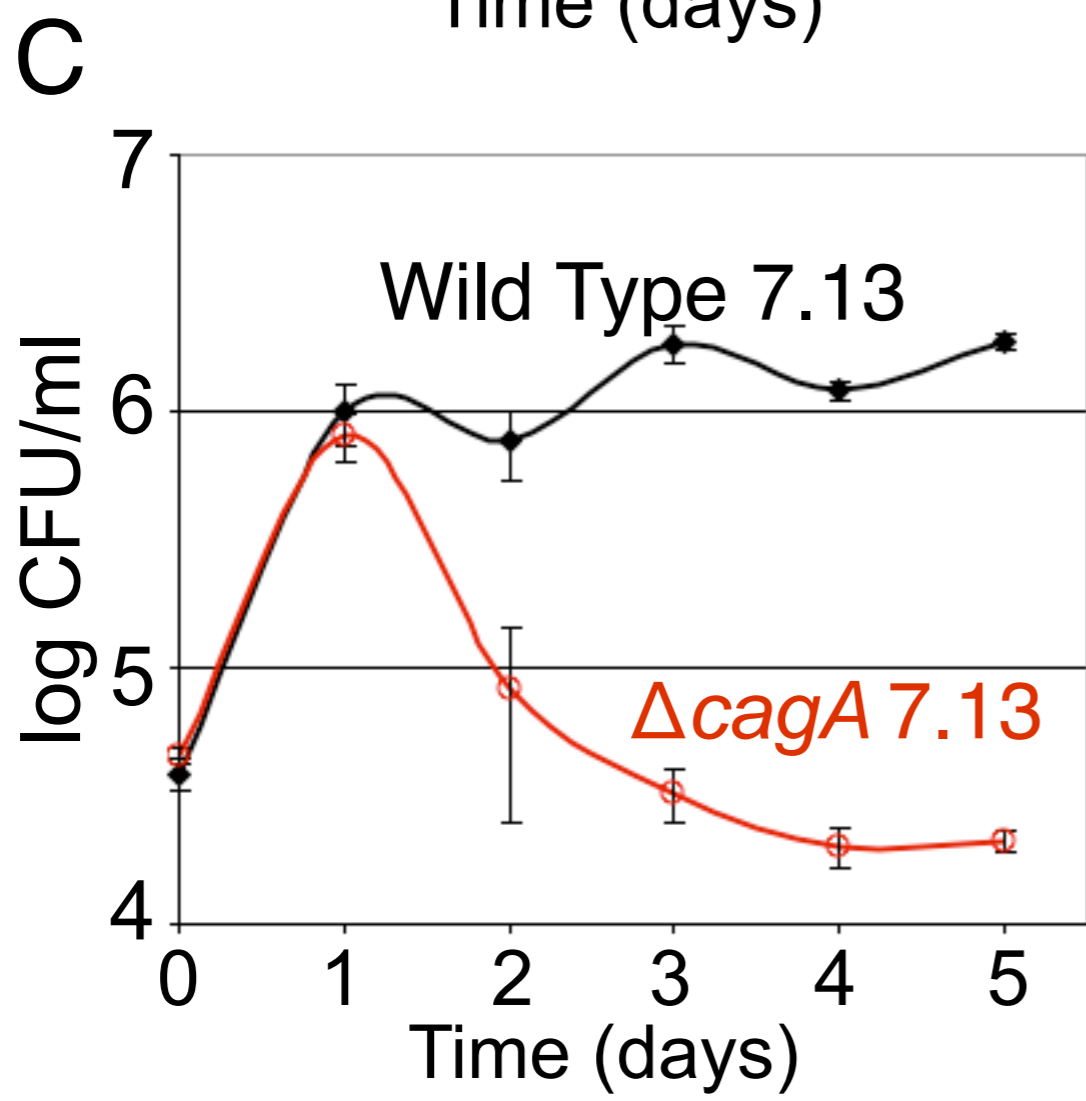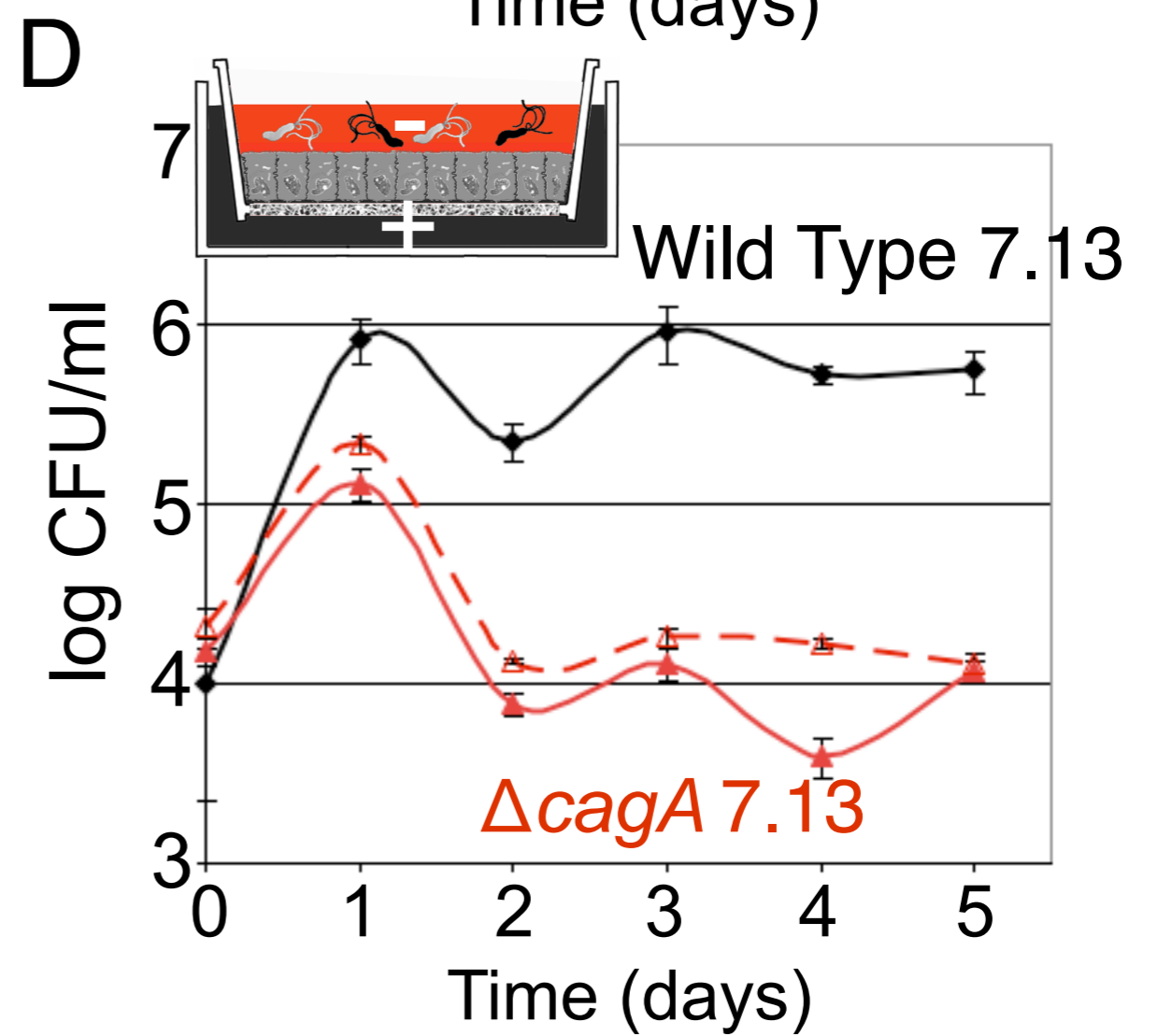

Supplement: Figure S2 — CagA is critical for Hp colonization of the apical cell surface. (A–C) Cells were infected in the Transwell system with strains indicated, and co-culture media added only to the basal chamber (+). DMEM was added to the apical chamber (−). Samples were taken from the apical chamber before wash daily, and plated for CFU counts. ΔcagE is a mutant defective in the ability to translocate CagA into host cells. ΔureAB is deleted for the ureA and ureB genes, marked with the same chloramphenicol resistance cassette as used in ΔcagA. 7.13 is an unrelated Hp strain, previously characterized for its ability to deliver CagA [27]. (D) ΔcagA is not rescued by WT in a mixed infection. 7.13 WT and ΔcagA were mixed together, and the mixture used to infect a monolayer on a Transwell filter. A monoculture of ΔcagA was also used to infect a separate Transwell filter (dashed line) at the same time. Samples were taken from the apical chamber before wash daily and plated on both non-selective and selective plates to differentiate WT and ΔcagA for CFU counts. (0.35 MB PDF) [file ppat.1000407.s003.pdf]

Figure S3

CagA delivery by reconstituted CagA\* *Hp* strain

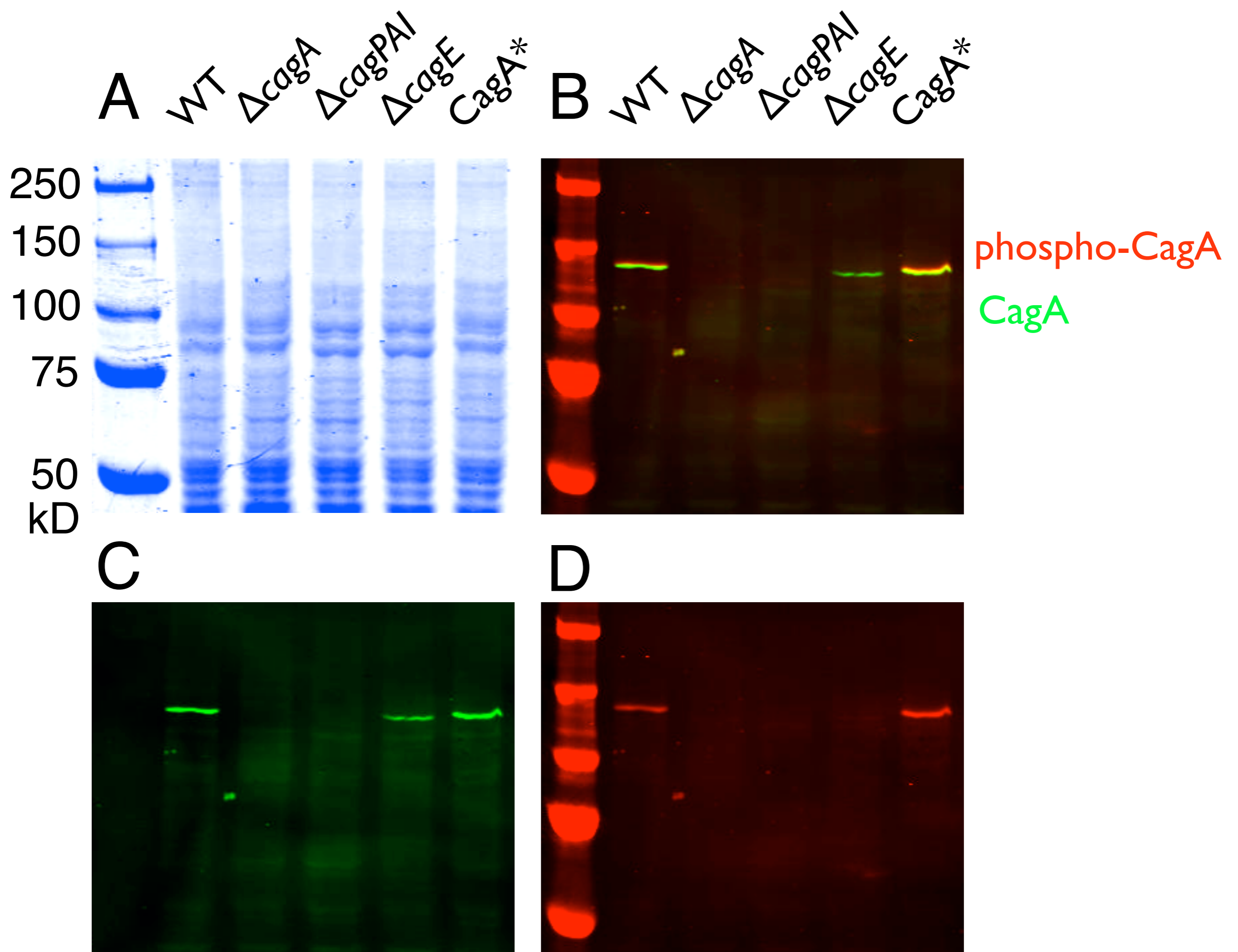

Supplement: Figure S3 — Reconstituted CagA° delivers equivalent amounts of CagA as WT. MDCK cells were infected with the indicated strains, and the infection allowed to proceed for 24 hours. Lysates from these infections were separated by SDS-PAGE, transferred to a nitrocellulose membrane, then immunoblotted with antibodies raised against the CagA-N-terminus (green, panel C) and against phosphorylated CagA (red, panel D). Panel B shows the merge of the anti-CagA-NT and anti-phospho-CagA blots. Panel A is a Coomassie Blue-stained gel showing total protein loaded. (0.25 MB PDF) [file ppat.1000407.s004.pdf]

Figure S4

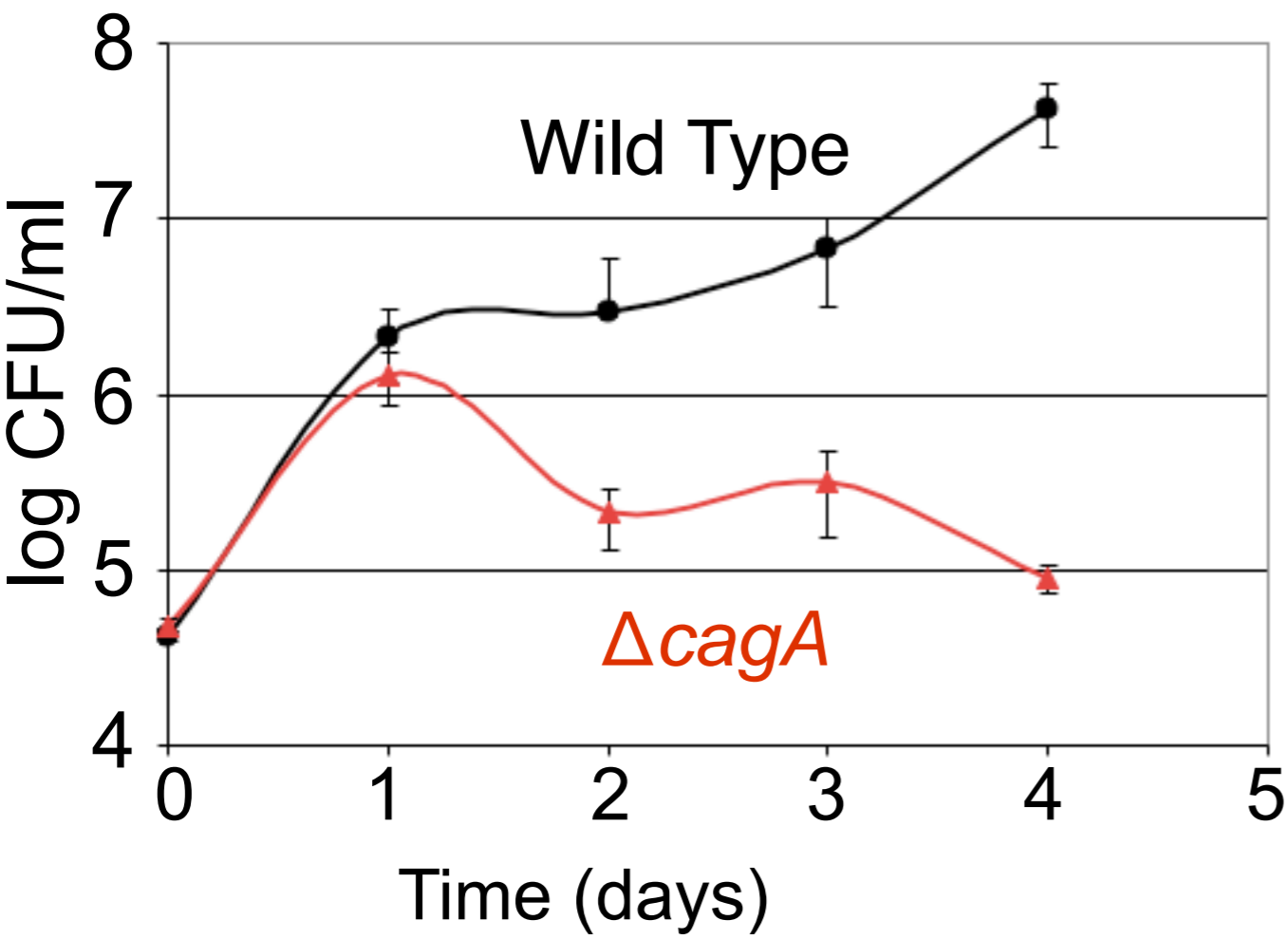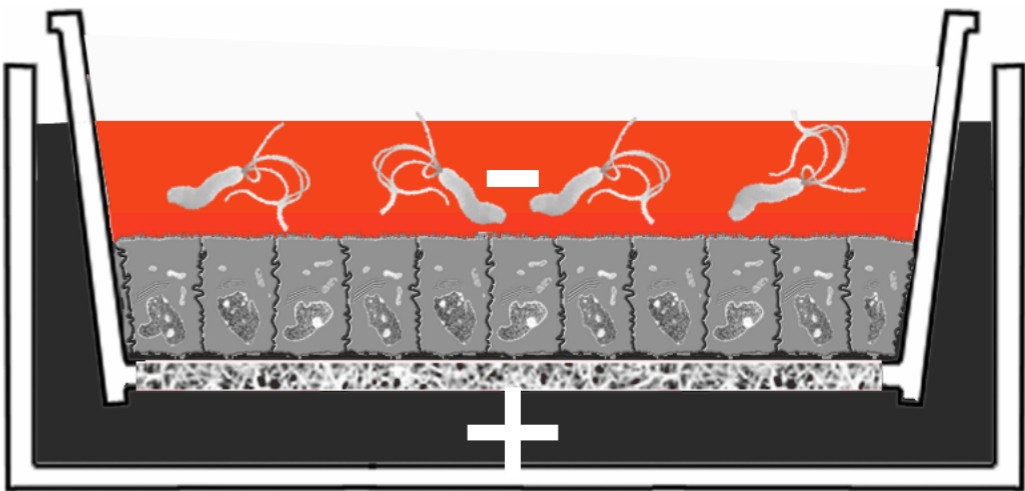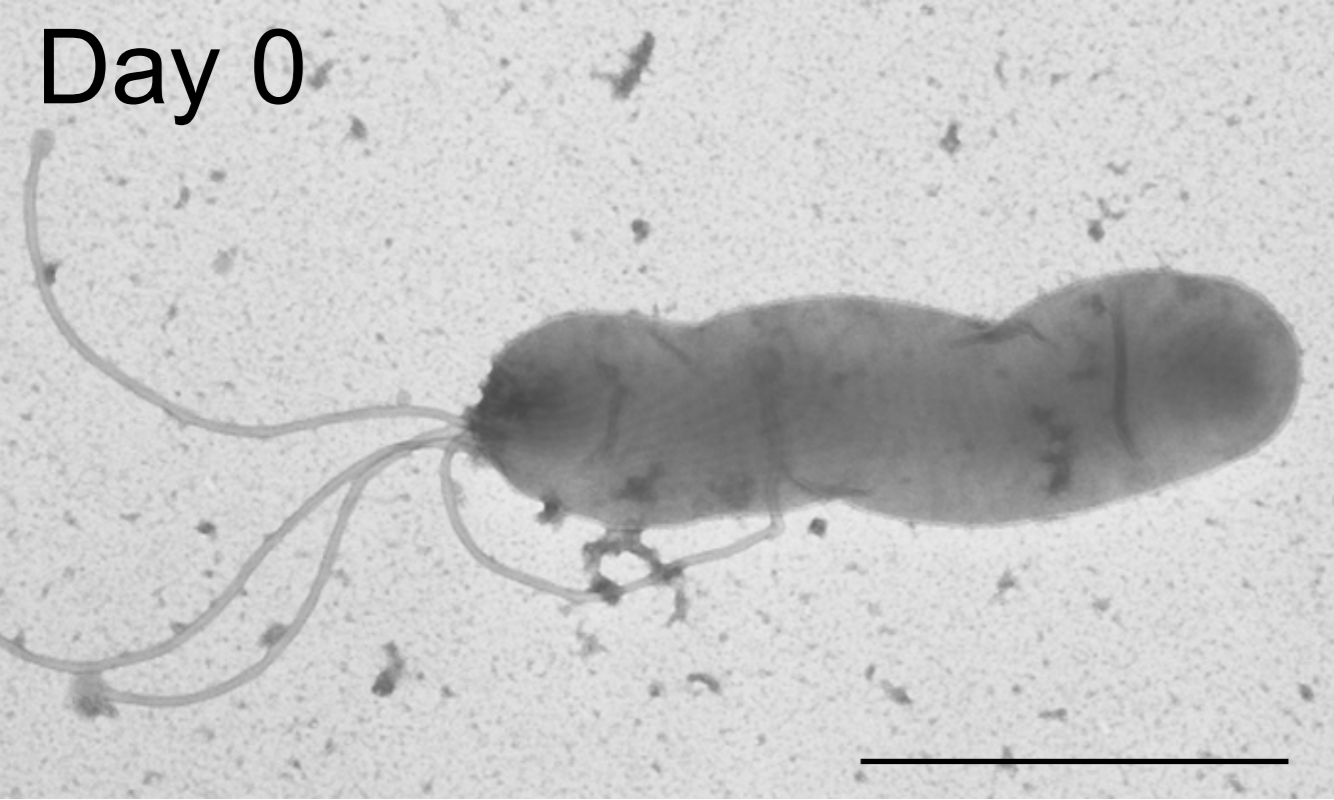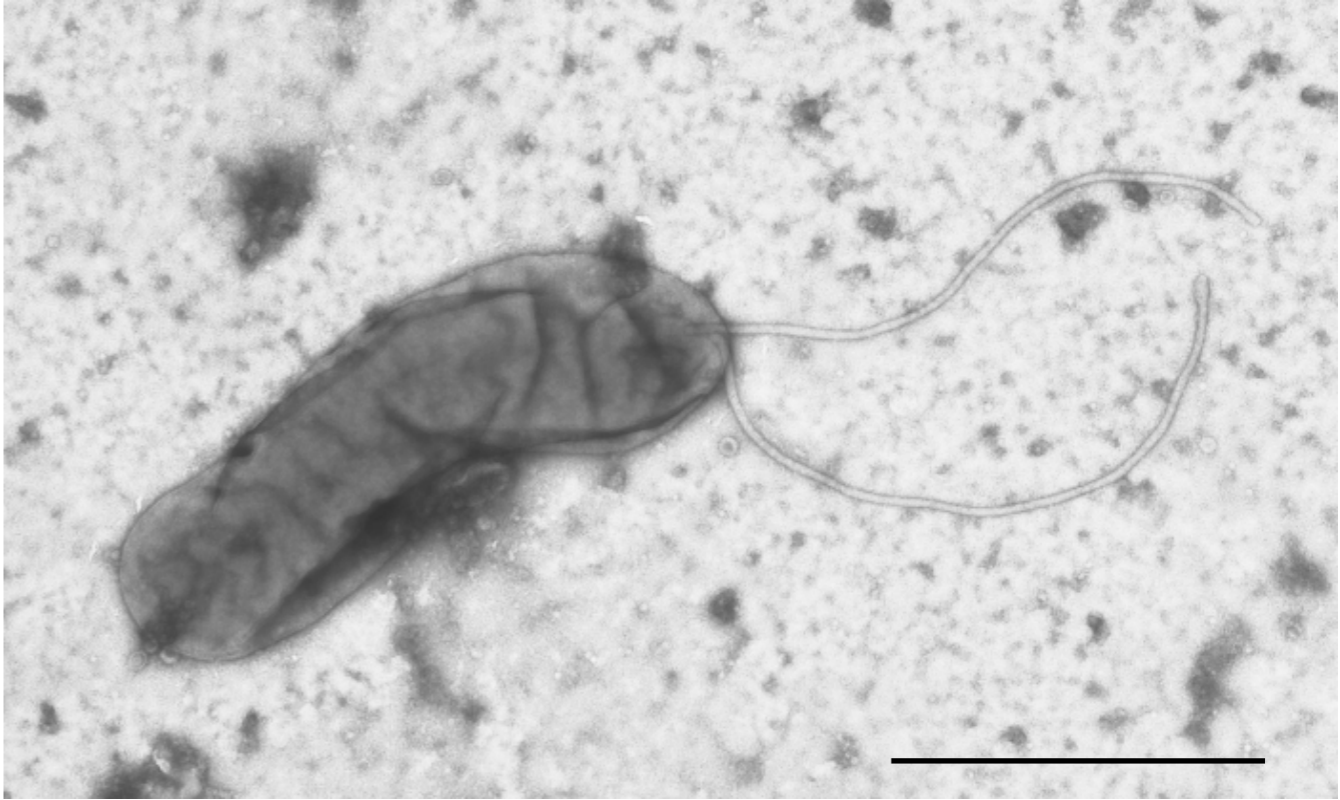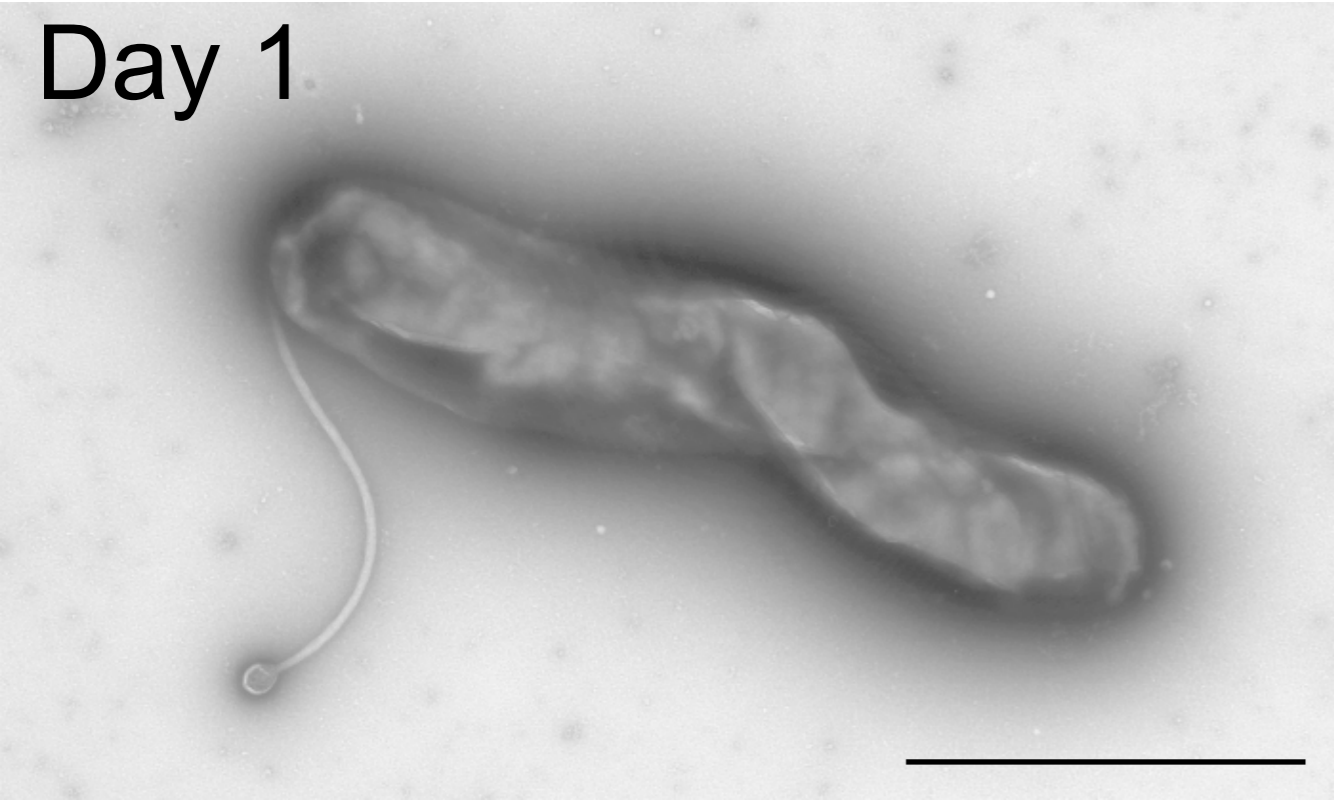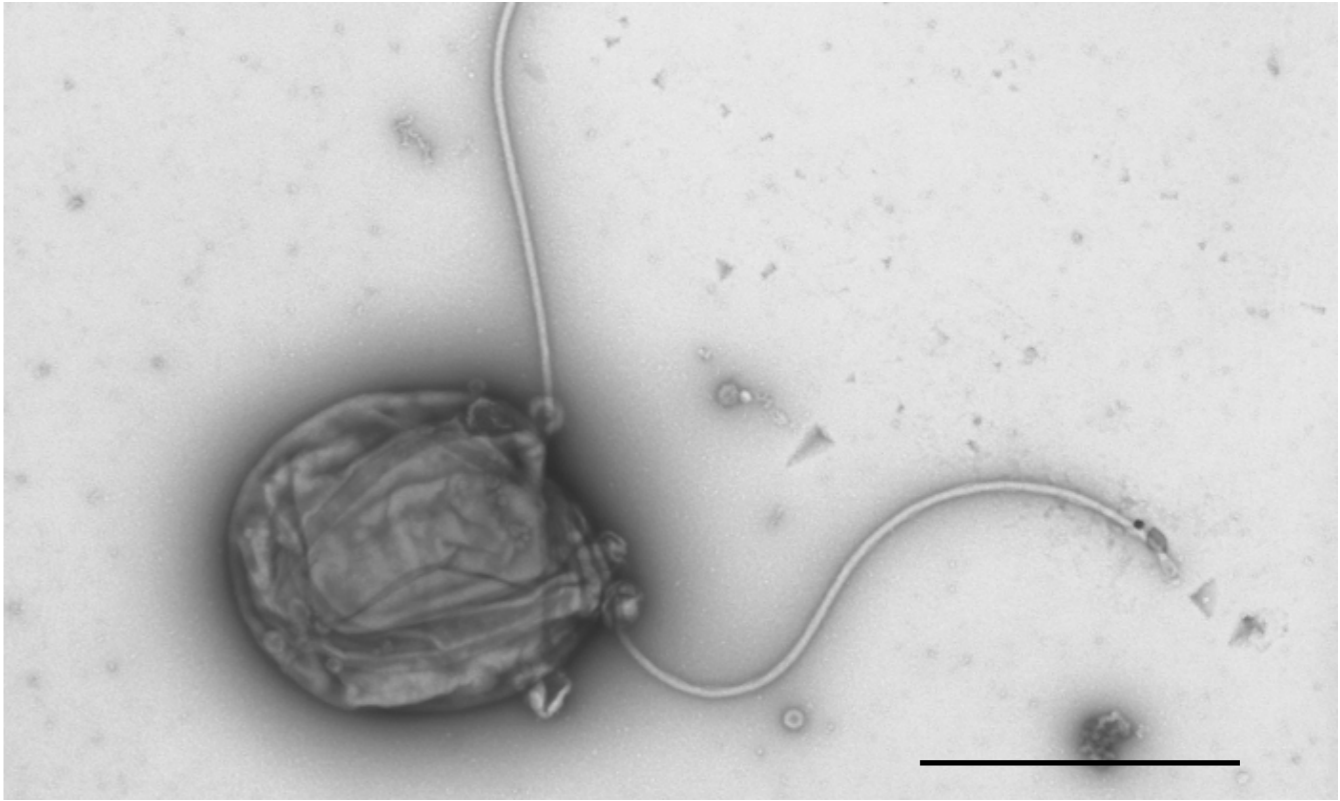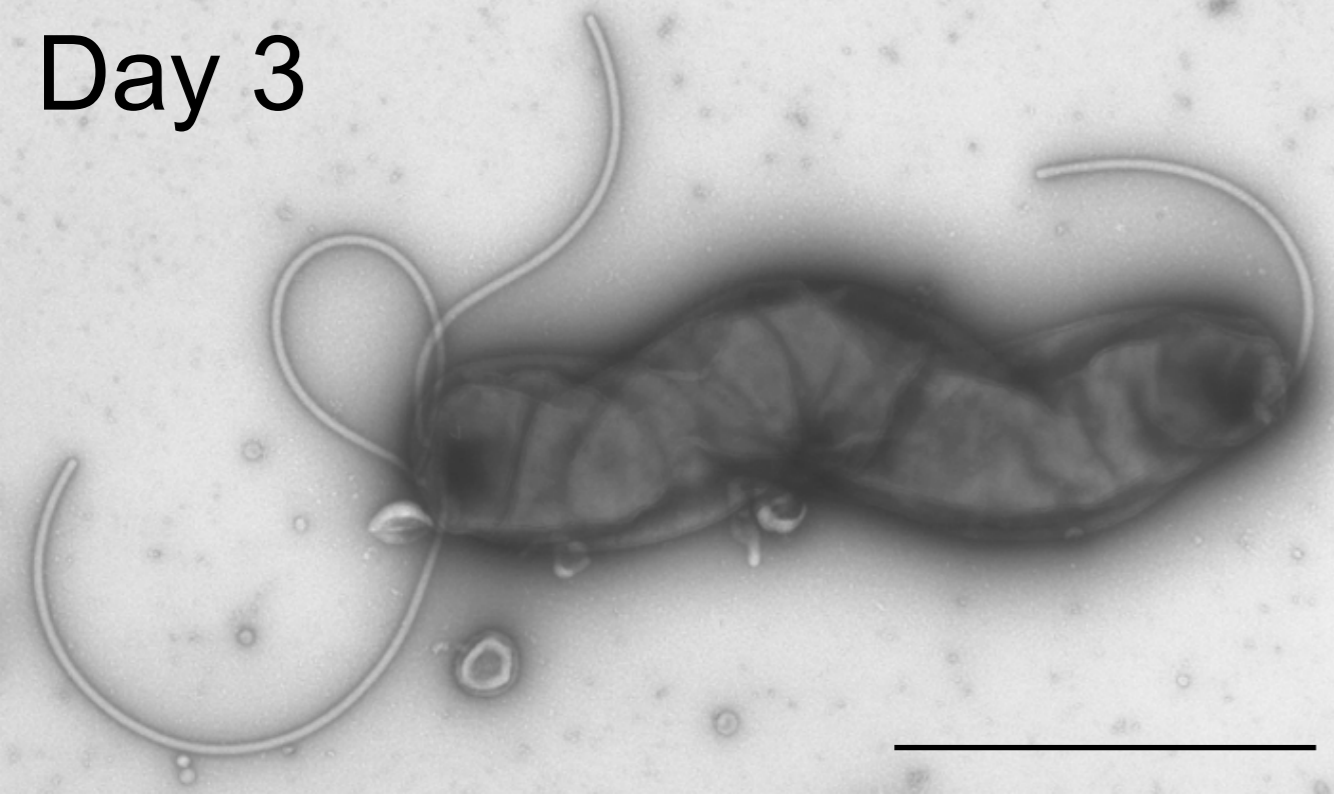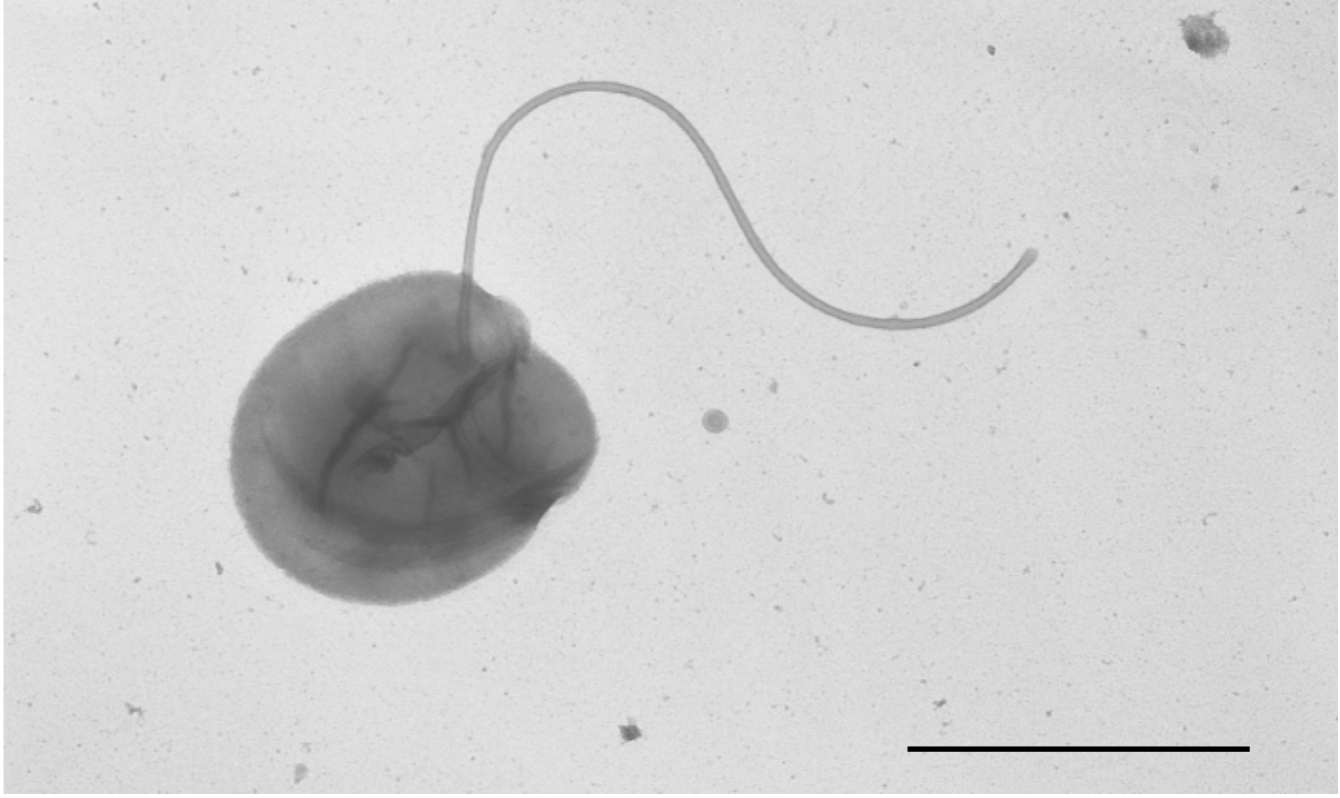

Wild Type

$\Delta cagA$

Supplement: Figure S4 — Change in morphology of free-swimming ΔcagA in the Transwell system. Polarized cells in the Transwell system were infected apically with WT or ΔcagA, and samples taken daily before wash for CFU counts (graph). At initial infection (Day 0), and at days 1 and 3 post-infection, samples were also taken from the apical chamber and the free-swimming bacteria examined by whole-mount TEM negative staining. Scale bars 1 µm. (1.65 MB PDF) [file ppat.1000407.s005.pdf]
